# Gut Microbiome Composition in Obese and Non-Obese Persons: A Systematic Review and Meta-Analysis

**DOI:** 10.3390/nu14010012

**Published:** 2021-12-21

**Authors:** Mariona Pinart, Andreas Dötsch, Kristina Schlicht, Matthias Laudes, Jildau Bouwman, Sofia K. Forslund, Tobias Pischon, Katharina Nimptsch

**Affiliations:** 1 Molecular Epidemiology Research Group, Max Delbrück Center for Molecular Medicine in the Helmholtz Association (MDC), 13125 Berlin, Germany; Mariona.PinartGilberga@mdc-berlin.de (M.P.); tobias.pischon@mdc-berlin.de (T.P.); 2 Department of Physiology and Biochemistry of Nutrition, Max Rubner-Institut (MRI)—Federal Research Institute of Nutrition and Food, 76131 Karlsruhe, Germany; andreas.doetsch@mri.bund.de; 3 Institute of Diabetes and Clinical Metabolic Research, University of Kiel, 24105 Kiel, Germany; Kristina.Schlicht@uksh.de (K.S.); matthias.laudes@uksh.de (M.L.); 4 Division of Endocrinology, Diabetes and Clinical Nutrition, Department of Internal Medicine 1, Kiel University, 24118 Kiel, Germany; 5 Microbiology and Systems Biology Group, Toegepast Natuurwetenschappelijk Onderzoek (TNO), Utrechtseweg 48, 3704 HE Zeist, The Netherlands; jildau.bouwman@tno.nl; 6 Experimental and Clinical Research Center, A Cooperation of Charité-Universitätsmedizin Berlin and Max Delbrück Center for Molecular Medicine, Lindenberger Weg 80, 13125 Berlin, Germany; Sofia.Forslund@mdc-berlin.de; 7 Charité-Universitätsmedizin Berlin, Corporate Member of Freie Universität Berlin, Humboldt-Universität zu Berlin, Berlin Institute of Health, 10117 Berlin, Germany; 8 Host-Microbiome Factors in Cardiovascular Disease Lab, Max Delbrück Center for Molecular Medicine in the Helmholtz Association (MDC), 13125 Berlin, Germany; 9 Structural and Computational Biology Unit, European Molecular Biology Laboratory, 69117 Heidelberg, Germany; 10 Biobank Core Facility, Berlin Institute of Health at Charité-Universitätsmedizin Berlin, 10178 Berlin, Germany; 11 German Centre for Cardiovascular Research (DZHK), Partner Site Berlin, 10785 Berlin, Germany; 12 Biobank Technology Platform, Max Delbrück Center for Molecular Medicine in the Helmholtz Association (MDC), 13125 Berlin, Germany

**Keywords:** gastrointestinal microbiome, adult, humans, obesity, BMI, 16S sequencing, shotgun metagenomics

## Abstract

Whether the gut microbiome in obesity is characterized by lower diversity and altered composition at the phylum or genus level may be more accurately investigated using high-throughput sequencing technologies. We conducted a systematic review in PubMed and Embase including 32 cross-sectional studies assessing the gut microbiome composition by high-throughput sequencing in obese and non-obese adults. A significantly lower alpha diversity (Shannon index) in obese versus non-obese adults was observed in nine out of 22 studies, and meta-analysis of seven studies revealed a non-significant mean difference (−0.06, 95% CI −0.24, 0.12, *I^2^* = 81%). At the phylum level, significantly more Firmicutes and fewer Bacteroidetes in obese versus non-obese adults were observed in six out of seventeen, and in four out of eighteen studies, respectively. Meta-analyses of six studies revealed significantly higher Firmicutes (5.50, 95% 0.27, 10.73, *I^2^* = 81%) and non-significantly lower Bacteroidetes (−4.79, 95% CI −10.77, 1.20, *I^2^* = 86%). At the genus level, lower relative proportions of *Bifidobacterium* and *Eggerthella* and higher *Acidaminococcus*, *Anaerococcus*, *Catenibacterium*, *Dialister*, *Dorea*, *Escherichia-Shigella*, *Eubacterium*, *Fusobacterium*, *Megasphera*, *Prevotella*, *Roseburia*, *Streptococcus*, and *Sutterella* were found in obese versus non-obese adults. Although a proportion of studies found lower diversity and differences in gut microbiome composition in obese versus non-obese adults, the observed heterogeneity across studies precludes clear answers.

## 1. Introduction

Obesity is a major public health problem worldwide [1]. In the year 2016, the global prevalence of overweight in adults (defined as body mass index, BMI ≥ 25 kg/m^2^) was 39% (1.9 billion adults), and the prevalence of obesity in adults (BMI ≥ 30 kg/m^2^) was 13% (650 million adults) [1]. It was estimated that by 2030, 2.16 billion (38%) individuals of the world’s adult population will be overweight and 1.12 billion (20%) will be obese [2]. Obesity is a risk factor for major chronic diseases, such as cardiovascular diseases [3], various types of cancer [4,5], and for premature death [6]. Obesity is the consequence of a positive energy balance resulting from the interaction of genetic and non-genetic factors, including personal, environmental, and nutritional factors [7]. There is evidence from animal studies that the gut microbiome may also play a role in the development of obesity [8,9]. Furthermore, some studies in humans suggest that the microbiome composition may differ between obese and non-obese persons, and it was speculated whether such differences may contribute to the higher disease risk observed in obese persons, but findings have been inconsistent [10].

The human gut microbiome is composed of two dominant phyla, Firmicutes and Bacteroidetes, accounting for 90% of the total community, as well as the phyla Proteobacteria, Actinobacteria, and Verrucomicrobia, which are less dominant [11]. Among the 200 different genera belonging to the Firmicutes phylum are *Lactobacillus*, *Bacillus*, *Clostridium*, *Enterococcus* and *Ruminococcus*, whereas the most predominant genera belonging to the Bacteroidetes phylum are *Bacteroides* and *Prevotella* [11], although taxonomic classifications depend on the reference database used [12]. Animal studies found that when gut microbiota from conventionally raised wild-type mice was transplanted into germ-free mice, a rapid increase in their body fat by 60% was observed without changing their food consumption [13], and when gut microbiota from obese adult female humans were transplanted to germ-free mice, a rapid increase in their body weight was also observed [14]. Another study showed that the gut microbiota of genetically obese mice (ob/ob) was less diverse compared to their lean counterparts, and was also found to be enriched in Firmicutes and depleted in Bacteroidetes [15]. In humans, findings have been inconsistent, with some studies finding a higher relative abundance of the Firmicutes as compared to the Bacteroidetes phyla (often also expressed as ratio) in obese as compared to non-obese persons, whereas others found no such associations [9,16,17,18,19,20,21]. Differences in the study population (e.g., age, sex, geographic region) or specific health-related subgroups (e.g., metabolic status in obese persons) could explain the high inter-individual variation in the gut microbial community of the gut microbiota [8], which precludes the definition of a reference microbiome in health and disease [22]. In addition, differences in microbiome measurement techniques and annotation are likely to have contributed to the heterogeneity of findings in the past [23].

Two systematic reviews exploring the differences in the gut microbiome composition between obese and non-obese mixing adults and children [24] or in adults only [25] have previously been conducted, although none conducted meta-analysis. Both these systematic reviews and other non-systematic reviews [26,27,28] tried to answer whether obesity is associated with a higher or lower gut microbiome diversity compared to lean individuals and also whether the Firmicutes to Bacteroidetes (F:B) ratio can be considered a relevant marker of gut dysbiosis in obese persons. The systematic reviews in adults from observational and intervention studies (bariatric surgery patients) [24,25] concluded that obese persons have different profiles of gut microbiota compared to the non-obese. Higher relative abundance of the phyla Firmicutes, Proteobacteria, and Fusobacteria, as well as the genus *Lactobacillus* were found in obese compared with lean individuals, whereas the phylum Bacteroidetes, as well as the species *Faecalibacterium prausnitzii*, *Akkermansia muciniphila*, *Methanobrevibacter smithii*, and *Bifidobacterium animalis* were found in lower relative abundance in the obese compared to the lean [25]. However, inconsistent results were found in the diversity of the gut microbiota associated with obesity [25]. Both systematic reviews attributed such inconsistent results to technical factors, such as the inclusion of studies that used different methodologies for the quantification of microorganisms [24,25]. Other technical factors that may contribute to divergent results are: different sequencing techniques for microbiome quantification, differences in the primers used and methods for DNA extraction, as well as the reference database used for the classification [23,25,29]. However, none of these systematic reviews addressed the issue of the lack of definition of criteria to select the appropriate reference database for taxonomic classification. Non-technical factors that have been shown to have an impact on the gut microbial composition, but are in available studies rarely or insufficiently controlled for include sex, season, dietary patterns, exercise, medication, and geographical aspects [25]. Both systematic reviews make a plea for future studies quantifying the gut microbiome using high-throughput sequencing techniques (so-called next generation sequencing technologies) to disentangle the complexity of the gut microbiome.

This is the first systematic review with meta-analysis addressing differences in the gut microbiome composition in obese versus non-obese adults focusing on studies using high-throughput sequencing technologies (e.g., 16S rDNA/rRNA sequencing, shotgun metagenomics). Our primary aim was to assess differences in the gut microbiome composition and diversity (alpha and beta-diversity) at the phylum and genus levels between obese and non-obese adult individuals from the general population based on data from observational studies. Further, we aimed to test the hypothesis that obese as compared to non-obese persons have a higher F:B ratio, which could be considered a hallmark of the pathophysiology of obesity. We also describe microbial taxonomic signatures associated with obesity reported by the included studies.

## 2. Materials and Methods

### 2.1. Data Sources and Search Strategy

This systematic review is reported in accordance with the recommendations set forth by the Preferred Reporting Items for Systematic Reviews and Meta-Analyses (PRISMA) statement [30]. The protocol was prospectively registered at PROSPERO (CRD42021225730). A systematic search was conducted in November 2020 using computerized bibliographic databases, such as PubMed and EMBASE via Ovid from inception to 19 November 2020. The search included a combination of MeSH terms and free text to retrieve articles on obesity, including anthropometric measurements, and gut microbiome, and was restricted to English language (see Supplement Table S1 for the search strategy).

We obtained further references from the reference list of the studies included in the qualitative synthesis. Search results were imported from PubMed and EMBASE XML into the CAMARADES Preclinical Systematic Review & Meta-analysis Facility (SyRF) (https://app.syrf.org.uk/ (last accessed on 5 March 2021)) for title and abstract screening as well as for full text assessment. To manage the references, we used EndNote X7^®^ (Thomson Reuters) software.

### 2.2. Exposures and Comparators

Obesity was the primary exposure of interest. Obesity was defined by body mass index (BMI) ≥ 30 kg/m^2^ as compared to non-obesity (BMI < 30 kg/m^2^). Secondary exposures were: (1) abdominal obesity based on waist circumference ≥102 cm in men and ≥88 cm in women, or waist-to-hip ratio ≥0.95 in men and ≥0.80 in women as compared to non-obesity based on waist circumference <102 cm in men and <88 cm in women, or waist-to-hip ratio <0.95 in men and <0.80 in women; (2) overweight individuals were defined by BMI ≥ 25 kg/m^2^ as compared to non-overweight (BMI < 25 kg/m^2^). Alternative BMI-based obesity definitions, e.g., for Asian populations, were also considered.

### 2.3. Outcome Measures

The primary outcomes of interest were: (1) differences in the alpha and beta-diversity between obese and non-obese persons; (2) differences in the Firmicutes to Bacteroidetes ratio between obese and non-obese persons; and (3) differences in gut microbiome composition between obese and non-obese persons. 

Microbial diversity can be expressed as the number of distinct species in a community (richness), the even distribution of their abundances (evenness) or a combination of both aspects, commonly termed alpha diversity. Microbial alpha diversity is estimated using the Shannon and Simpson indices, whereas microbial richness is estimated using the Chao1 index, or number of observed species/operational taxonomic units (OTUs).

The secondary outcome of interest was to describe microbial taxonomic signatures associated with obesity.

### 2.4. Inclusion and Exclusion Criteria

Inclusion criteria were human observational studies comparing the composition of the gut microbiome between obese and non-obese adult individuals (age ≥ 18 years) recruited from the general population, irrespective of ethnicity. If studies included a mixed population of children and adults, only those studies presenting their data for adults separately were included. A further inclusion criterion was that the gut microbiome was measured by means of high-throughput analyses (e.g., 16S rDNA/rRNA sequencing, shotgun metagenomics) in fecal samples.

Exclusion criteria were intervention studies and randomized controlled trials. Further exclusion criteria were: (1) no report of data on obese and non-obese persons, or (2) gut microbiome measured in samples other than feces or by means of culture-dependent techniques or other non-high-throughput sequencing techniques, or (3) in adults that have undergone different types of bariatric surgery. Studies focusing on specific diseases, written in a language other than English, or published as abstract, editorial or comment were also excluded.

Systematic and narrative reviews were kept for background only and their data were not extracted. 

### 2.5. Study Selection and Data Extraction

Two researchers (MP and KN) independently screened the titles and abstracts, and reviewed the full texts. Disagreements were resolved through discussion and consensus, or if necessary, with referral to a third researcher (TP). The same investigators designed and piloted a data extraction form before routine use, and extracted the data independently. When needed, the primary study author was contacted to obtain further information. For each included study the following information was extracted: study ID (first author and year of publication), country, study design, study population, sample size, definition of obesity, overweight and non-obesity, method used to measure microbiome, DNA extraction method, platform used, outcomes assessed, results on composition and diversity of gut microbiome in obese versus non-obese and/or differences in comparison groups, and characterization of microbiome taxonomic signatures in obese persons. We also selected relevant titles from the reference lists of those studies that were finally included for qualitative synthesis, obtained their abstract and followed the same procedure as above described to identify further studies.

### 2.6. Quality Assessment

Two independent researchers (MP and KN) appraised the risk of bias using an adapted version of the Risk Of Bias In Non-Randomized Studies of Interventions (ROBINS-I) tool [31], a tool proposed by Cochrane that can be also applied to appraise the risk of bias in observational studies. Discrepancies were resolved by discussion and consultation with a third author where necessary. The adapted version of ROBINS-I tool comprised six domains of bias due to: (1) confounding, (2) selection of participants, (3) exposure assessment, (4) missing data, (5) measurement of the outcome, and (6) selective reporting of the results, together with the signaling questions that facilitated the judgement of potential risk of bias for each domain as described in Supplement Table S2. The overall judgment of risk of bias was categorized as low, moderate or serious as previously described [31]. If at least one domain was judged to be of serious risk but not at critical risk in any other domain, then the overall judgment of the risk of bias was deemed as serious. If all domains were rated as being at low risk of bias, then the overall judgment was deemed as low. If all domains were rated at low or moderate risk of bias, then the overall judgement was deemed as moderate.

### 2.7. Data Synthesis

For the data synthesis studies that presented results separately for different subpopulations defined by sex [32,33] or geographic region [34,35] were counted as separate studies. 

For a qualitative data synthesis, we counted the number of studies addressing each outcome that showed significantly higher or lower or non-significant differences for the respective outcome in obese versus non-obese adults as reported by the studies and/or based on nominal *p*-values (*p* < 0.05 considered statistically significant). 

For the quantitative data synthesis, we used the extracted study-specific means and nominal *p*-values or the mean difference (MD) between obese and non-obese adults with 95% confidence intervals (CIs) to combine multiple studies that measured the same continuous outcome using similar methods and the same unit of measurement. We also extracted the means and standard deviation (SD) to calculate the MD 95% CIs for the meta-analysis. If standard deviation data were not available, it was calculated based on *p* values or standard errors, if possible (https://training.cochrane.org/resource/revman-calculator (accessed on 27 July 2021)). Because we anticipated substantial between-study heterogeneity, a random-effects model was applied to obtain pooled effect size estimates, 95% CIs and *p*-values through the inverse variance method and restricted maximum-likelihood estimator [36]. Statistical heterogeneity was determined by the *I^2^* statistic, and it was regarded as substantial if *I^2^* was found to be between 40% and 70%. A sensitivity analysis was performed when estimates revealed heterogeneity >70% by removing one study at a time, and the study driving the heterogeneity was excluded from the meta-analysis. Because fewer than 10 studies for each outcome qualified for meta-analyses, publication bias was not inspected. The “meta” (Version 4.18-0) and “metaphor” (Version 2.4-0) packages were used to perform meta-analyses in R and statistical significance was determined at the *p* < 0.05 level.

## 3. Results

### 3.1. Search Flow and Studies Overview

**Figure 1:**
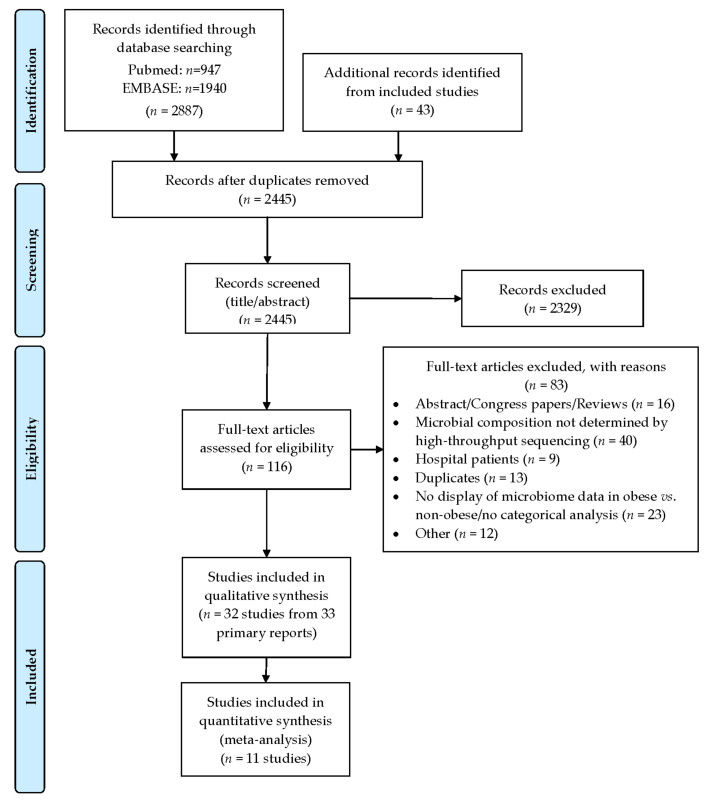
Description of the selection of the included studies following a PRISMA flow diagram.

The detailed output of the searches and the process followed for the identification is displayed in the PRISMA flowchart (Figure 1). From a total of 2445 unique hits, 116 studies were selected for full-text review. Of these, 33 primary reports [17,18,19,20,21,32,33,34,35,37,38,39,40,41,42,43,44,45,46,47,48,49,50,51,52,53,54,55,56,57,58,59,60], including 32 studies met the criteria for inclusion and were selected for data extraction and synthesis. 

**Figure 2:**
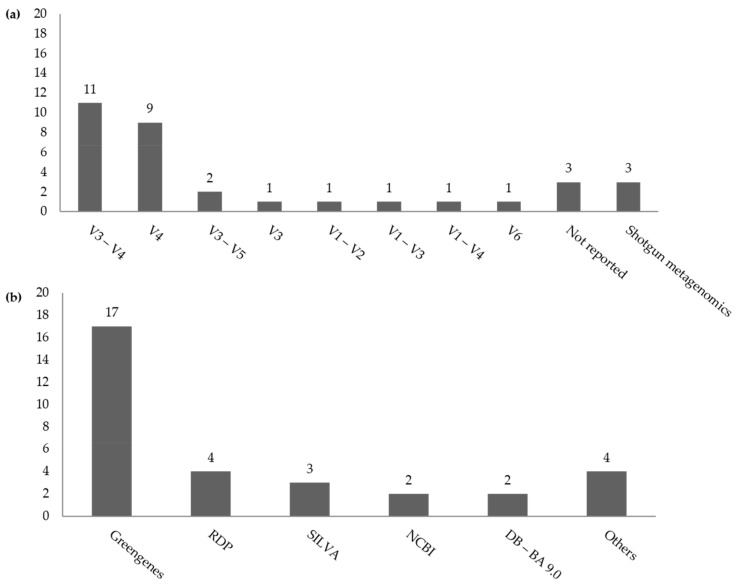
SrRNA, by amplified region, or Shotgun metagenomics; (**b**) reference databases used for taxonomic classification reported in 32 studies. Note that some studies amplified multiple regions or used multiple databases.

Table 1 describes the characteristics of the included studies, which were of cross-sectional design and were published between 2012 and 2020, yielding 13,186 individual stool samples for microbiome analyses. All studies included both males and females with the exception of two studies [19,34] that included females only and one study [35] that included males only. Ten studies were from North and South America (Canada, *n* = 1; Mexico, *n* = 1; USA, *n* = 6; Brazil, *n* = 1; Colombia, *n* = 1), nine were from Asia-Pacific countries (Australia, *n* = 1; Bangladesh, *n* = 1; China, *n* = 1; India, *n* = 1; Japan, *n* = 3; Korea, *n* = 1; Saudi Arabia, *n* = 1), seven from Europe (Finland, *n* = 2; Germany, *n* = 1; Italy, *n* = 2; Netherlands, *n* = 1; UK, *n* = 1), two from Africa (Egypt, *n* = 1; South Africa, *n* = 1), and four were conducted in multiple countries (Table 1). Two studies also assessed the fungal composition and diversity of the human gut microbiota in obese and non-obese adults [38,48]. The region of amplification of the 16S rRNA gene varied across studies, such as such V3–V4 (*n* = 11), V4 (*n* = 9), V3–V5 (*n* = 2), V3 (*n* = 1), V1–V2 (*n* = 1), V1–V3 (*n* = 1), V1–V4 (*n* = 1), and V6 (*n* = 1). Three studies [18,56,59] did not specify the amplified region of the 16S rRNA gene (Figure 2). To explore the fungal composition and diversity of the gut microbiota, one study amplified the fungal 18S rRNA gene (region V7–V8) with fungal primers [38], and another study amplified the ITS1 fragment [48]. The most widely used sequencing platform was the Illumina MiSeq (*n* = 23). Shotgun metagenomics was conducted in three studies that used BaseSpace through the 16S Metagenomics app from Illumina^®^ [45], Illumina HiSeq [55], and the Ion-Proton sequencing platforms [57]. Two studies used high-throughput methods that are not considered as next-generation sequencing, but are the microarray-based HITChip method [56] and parallel Sanger sequencing of cloned amplicons of the 16S rRNA gene [52]. The database in which taxonomic binning of 16S reads were based on was not reported in three studies [32,40,53]. The rest of the studies mainly used the Greengenes database (*n* = 17) [18,19,37,39,41,43,44,45,46,48,50,51,54,58,59,60], RDP (*n* = 4) [20,34,52,55], SILVA (*n* = 3) [35,47,54], NCBI (*n* = 2) [38,57], or DB-BA9.0 (*n* = 2) [17,21]. Other databases used were: Broad Institute Microbiome Utilities microbiomeutil-r20110519 database [33], KEGG (Kyoto Encyclopedia of Genes and Genomes) Orthology (KO) Database at level 2 and level 3 [54], HUMAnN2 v0.11.1 [55] for shotgun metagenomics, and UNITE reference database for fungal taxonomy [48] (Figure 2). One study did not use a publicly available database but a HITChip specific database [56]. 

A total of two studies used BMI < 30 as reference [20,57], nine studies used BMI < 25 [18,26,34,35,43,44,47,53,55], one study used BMI < 20 [21], fifteen studies used BMI 18.5–24.9 [19,32,33,41,45,46,48,49,51,52,54,56,58,59,60], and one study from Japan used BMI < 18.5 as a reference [17]. However, in general, studies from Asia used lower cut-offs than studies from Europe to define obesity. Three other studies did not specify the BMI cut-offs of comparison groups [9,37,38]. One study did not use the BMI but instead used the fat mass index (FMI) [39], and two studies used both the BMI and the visceral fat area (VFA) [32] or the waist circumference [43].

### 3.2. Primary Outcomes

#### 3.2.1. Alpha and Beta Diversity (Microbial Diversity and Richness, Microbial Dissimilarities)

**Figure 3:**
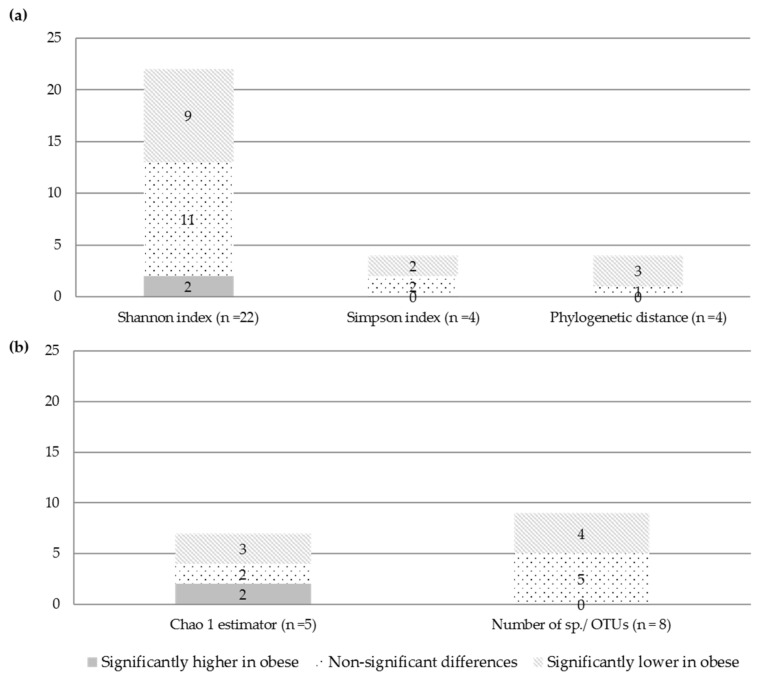
Number of studies that reported alpha diversity indices Panel (**a**) or richness estimators Panel (**b**) as significantly higher (grey), lower (diagonal stripes) or not different (dotted) when comparing obese to non-obese persons.

Of the 32 included studies, 25 studies investigated alpha diversity in obese versus non-obese adults, two did not assess alpha diversity, and five studies did not report alpha diversity stratified by BMI (Table 2). At the individual study level, results on the difference in alpha diversity between obese and non-obese persons were discrepant (Figure 3): the Shannon index, reported in 22 studies, was found to be significantly lower in obese compared to non-obese adults in nine studies [17,32,35,37,41,46,48,51,54], higher in two studies [21,54], and not significantly different in eleven studies [19,33,34,35,39,44,45,47,49,52,53]. The study of Salah et al. [54] found significantly lower diversity (Shannon index) among obese as compared to non-obese persons and higher diversity among obese diabetic compared to non-obese persons. Yasir et al. [35] found significantly lower diversity (Shannon index) among obese as compared to non-obese persons only among the French participants, but no significant differences among the Saudi Arabian participants. In order to further explore the role of methodological factors in microbiome measurement, we restricted the qualitative analysis of the Shannon index (*n* = 22 studies in total) to studies that amplified the V3–V4 region (most commonly amplified region, *n* = 12 studies), studies that used the Greengenes database (most commonly used database, *n* = 10 studies) as well as studies that amplified the V3–V4 region and used Greengenes database (*n* = 3 studies) (Supplement Figure S1). Restriction to studies amplifying the V3–V4 region revealed that half of the studies (six out of twelve) observed a lower Shannon index in obese versus non-obese persons and restriction to studies amplifying the V3–V4 region and using the Greengenes database revealed that a fifth of the studies (two out of ten) observed a lower Shannon index in obese versus non-obese persons (Supplement Figure S1).

The Simpson index, reported in four studies, was found to be significantly lower in obese compared to non-obese persons in two studies [46,56] and to be non-significantly different in two studies [19,52]. Another four studies used the phylogenetic distance (PD) between samples to assess diversity and it was found to be significantly lower in obese than non-obese persons in three studies [18,55,60] and non-significant in one study [49]. 

The Chao1 estimator for richness, reported in seven studies, was found to be lower in obese compared to non-obese persons in three studies [35,39,51], and higher in obese persons in two studies [19,34], and non-significant in two studies for the populations of Saudi Arabia [35] and Soweto [34]. The number of observed species/OTUs, reported in nine studies, was found to be lower in obese compared to non-obese persons in four studies [33,35,39,41], and non-significantly different in five studies [35,44,46,49,51]. Peters et al. [33] observed significantly lower richness in obese versus non-obese women (*p* = 0.03), but not in men (*p* = 0.47), and Yasir et al. [35] found a significantly lower number of OTUs among obese as compared to non-obese persons only among the French, but no significant differences in richness between obese and non-obese persons among the Saudis (Figure 3). One study [40] used a Shannon index Effective Number of Species to measure richness and found no significant differences between obese and non-obese persons.

**Figure 4:**
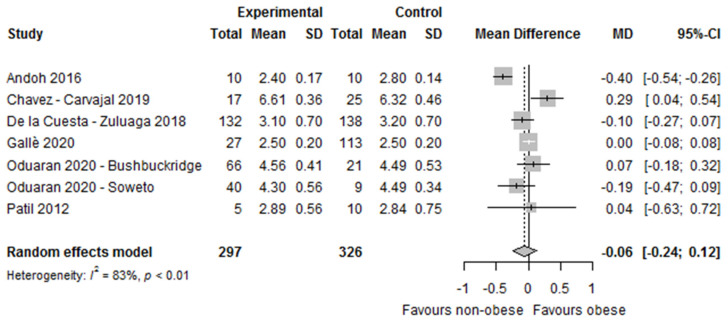
Forest plot of the differences in alpha diversity between obese and non-obese persons by Shannon index. Study references: Andoh et al. [17], Chavez-Carvajal et al. [19], De la Cuesta—Zuluaga et al. [41], Gallè et al. [45], Oduaran et al. [34], and Patil et al. [52].

Of the 22 studies that used the Shannon Index as an alpha diversity measure to compare obese and non-obese persons, only seven studies provided sufficient information for meta-analysis. The forest plot (Figure 4) showed that differences in microbial diversity between obese and non-obese persons, as assessed by the Shannon index, did not reach statistical significance. Given the substantial heterogeneity, we conducted sensitivity analysis by omitting one study at a time. These analyses suggested that one study from Japan (DB-BA 9.0 taxonomic reference database used), which observed a significantly lower Shannon Index in obese versus non-obese persons, contributed the most to heterogeneity in the meta-analysis on the Shannon index [17]. However, when we excluded this study from the analyses, the mean difference between obese and non-obese persons changed direction, but remained statistically non-significant (MD 0.01, 95%CI −0.11, 0.13, *I^2^* = 43%, *n* = 6 studies, Supplement Figure S2).

A total of 24 studies assessed beta diversity, although nine studies did not report beta diversity by BMI. Eleven studies [17,19,21,33,34,35,49,52,54,55,60] found microbial dissimilarities across the BMI groups, whereas only four studies did not find any [18,45,46,53].

#### 3.2.2. Differences in The Microbial Composition

##### At the Phylum Level

Differences in the relative abundance of the gut microbial composition at the phylum level between obese and non-obese persons are depicted in Table 3. 

**Figure 5:**
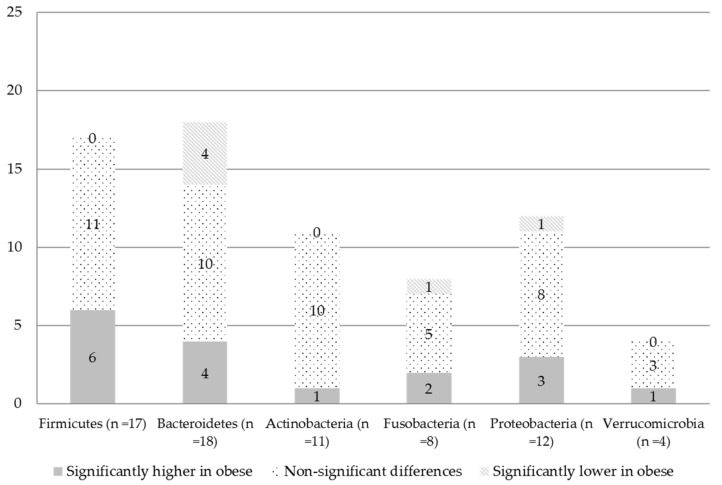
Number of studies that reported differences in the relative abundance of phyla as significantly higher (grey), lower (diagonal stripes) or not different (dotted) when comparing obese to non-obese persons.

**Figure 6:**
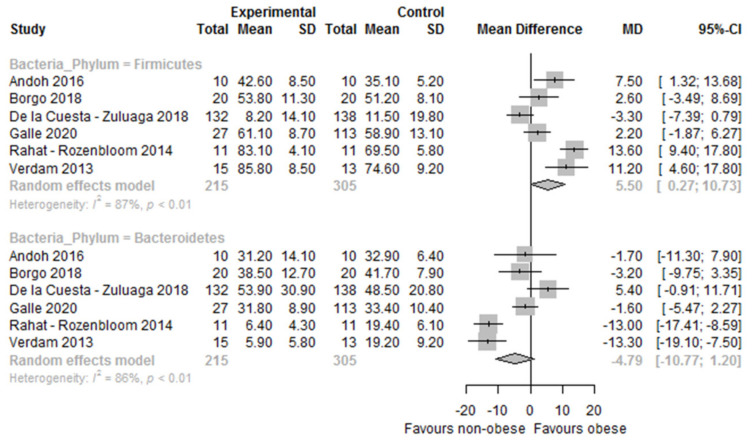
Forest plot of the differences in the gut microbiome composition at phylum level between obese and non-obese. Study references: Andoh et al. [17], Borgo et al. [18], De la Cuesta—Zuluaga et al. [41], Gallè et al. [45], Rahat—Rozenbloom et al. [53], and Verdam et al. [56].

At the individual study level, regarding Firmicutes, out of 17 studies, 11 studies did not observe statistically significant differences in their relative abundance between obese and non-obese persons [18,20,21,33,34,41,44,45,46,52,54], whereas six studies observed significantly higher proportions in the relative abundance in the obese group compared to the non-obese group [17,19,32,35,53,56] (Table 3 and Figure 5). The study of Yasir et al. [35] found significantly higher relative abundance of Firmicutes in the obese group compared to the non-obese only in the sample from Saudi Arabia (men only) but not in the French study arm (men and women). Interestingly, Ozato et al. [32] found higher proportions of Firmicutes in women with a higher BMI (*p* for trend: 0.004) but not in men. Differences in the relative abundance of Bacteroidetes showed contradictory results (Table 3). Out of 18 studies, 10 studies did not observe statistically significant differences between obese and non-obese persons [17,18,19,20,33,44,45,52,53,54], four studies found a higher relative abundance of Bacteroidetes in obese persons compared to non-obese persons [34,35,46,49], whereas four studies found lower relative abundance of Bacteroidetes among obese persons compared to non-obese persons [21,32,41,56] (Figure 3). The study of Ozato et al. [32] found a significant decrease in the relative abundance of the Bacteroidetes phylum across BMI groups in women (*p* for trend: <0.001) but not in men. The study of Yasir et al. [35] found significantly higher proportions in the relative abundance of Bacteroidetes in French obese persons compared to their non-obese counterparts (including both men and women). Differences in the relative abundance between obese and non-obese persons for the phyla of Firmicutes and Bacteroidetes are also depicted in Figure 5. Study-level meta-analyses could only be performed for the phyla of Firmicutes and Bacteroidetes in six studies (Figure 6). Sensitivity analysis were performed by omitting one study at a time, but the heterogeneity was still substantial (*I^2^* > 70%). 

The forest plot showed a statistically significant higher relative abundance of Firmicutes in obese compared to non-obese persons (MD 5.50 [95%CI 0.27, 10.73]; *I^2^* = 87%, *n* = 6) but non-significantly lower Bacteroidetes (MD −4.79 [95%CI −10.77, 1.20]; *I^2^* = 86%, *n* = 6) (Figure 4). 

**Figure 7:**
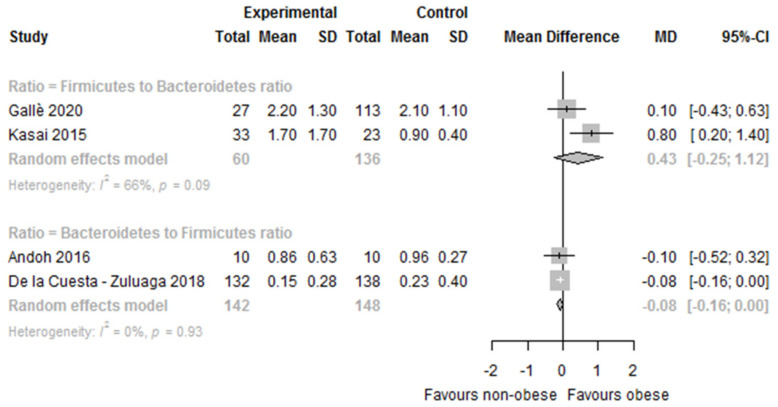
Forest plot of the differences in the Firmicutes to Bacteroidetes ratio between obese and non-obese. Study references: Andoh et al. [17], De la Cuesta—Zuluaga et al. [41], Gallè et al. [45], and Kasai et al. [21].

Ten studies reported data on the F:B ratio, although meta-analysis could only be conducted in two studies. Three studies reported data on the B:F ratio and meta-analysis could be performed in two of the three studies (Table 3). The results were controversial: four studies reported significant differences between obese and non-obese persons (higher F:B ratio in obese than in non-obese persons) [19,21,48,53], while six studies reported no significant differences [33,45,49,54,58,60]. In the meta-analysis, we found a statistically non-significantly higher F:B ratio in obese versus non-obese persons (MD 0.43 [95%CI −0.25, 1.12]; *I^2^* = 66%, *n* = 2). Regarding the B:F ratio, two studies reported significant differences between obese and non-obese persons (B:F ratio lower in obese than in non-obese) [41,56], while one study reported no significant differences in the B:F ratio [17]. In the meta-analysis, a statistically significantly lower B:F ratio in obese versus non-obese persons (MD −0.08 [95%CI −0.16, −0.00]; *I^2^* = 0%, *n* = 2) was observed (Figure 7).

Differences in the relative abundance between obese and non-obese persons for the phyla of Actinobacteria (*n* = 11 studies), Fusobacterium (*n* = 8), Proteobacteria (*n* = 12) and Verrucomicrobia (*n* = 4) are depicted in Table 3 and Figure 5. Regarding Actinobacteria (*n* = 11), none of the studies found differences between the groups, with the exception of one study that found a higher abundance of Actinobacteria in obese persons compared to the rest of the groups (normal weight, diabetes, and obese with diabetes) (*p* = 0.04) [54]. Regarding the phylum Fusobacteria (*n* = 8), five studies found no differences in their relative abundance between obese and none obese persons [19,20,21,34,54], whereas two found a higher proportion [17,46], and one found a lower proportion of Fusobacteria in obese persons compared to the non-obese [47]. Regarding the phylum of Proteobacteria (*n* = 12), eight studies did not observe statistically significant differences between obese and non-obese persons [17,18,19,21,32,34,44,52], three found higher proportions of Proteobacteria in obese persons [35,46,56], and one found lower proportions [54]. The study of Yasir et al. [35] found significantly higher proportions in the relative abundance of Proteobacteria in French obese persons compared to their non-obese counterparts (including both men and women), whereas no significant differences were observed in Saudi obese persons compared to their non-obese counterpart (men only). Regarding the phylum Verrucomicrobia (*n* = 4), three studies found no differences in their relative abundance between obese and non-obese persons [18,34,35], whereas one study found a higher proportion of Verrucomicrobia in obese compared to non-obese persons [54].

##### At the Genus Level

A total of 27 studies reported data on the relative abundance between obese and non-obese persons at the genus level. A list of bacteria found to be statistically significantly higher or lower in obese compared to non-obese persons is depicted in Table 4. 

*Significantly higher in obese*

Thirteen genera, namely, Acidaminococcus, Anaerococcus, Catenibacterium, Dialister, Dorea, Escherichia-Shigella, Eubacterium, Fusobacterium, Megasphera, Prevotella, Roseburia, Streptococcus, and Sutterella were found to be significantly more abundant among obese persons than among the non-obese in at least two studies. 

*Significantly lower in obese*

*Bifidobacterium* and *Eggerthella* were found to be significantly less abundant among obese persons than among the non-obese in at least two studies. 

*Controversial findings*

Discrepant results were found in 13 genera of the bacteria Akkermansia, Alistipes, Anaerotruncus, Bacteroides, Blautia, Clostridium, Coproccocus, Desulfovibrio, Faecalibacterium, Oscillibacter, Oscillospira, Parabacteroides, and Ruminoccocus where they have been found in higher relative abundance in obese persons in some studies and in lower relative abundance in others, compared to non-obese persons (Table 4).

##### Fungi

Only two studies investigated differences in the mycobiota composition between obese and non-obese persons. The study of Borges et al. [38] found that *Penicillium* sp., *Paecilomyces* sp., and *Fonsecaea* sp. fungi separated the obese group from the other groups (overweight and normal weight). A slightly higher diversity was found in normal weight persons, and *Syncephalastrum* sp. (Zygomycota), which was found only in normal weight persons. *Paecilomyces* sp. was predominant in normal weight and overweight persons, whereas *Penicillium* sp. was the most frequently identified fungi in the obese group. Furthermore, obese persons displayed higher yeast counts. Whilst *Trichosporon* sp. was the most prevalent yeast in normal weight persons, *Rhodotorula* was increased in overweight and obese persons. The study of Kaplan et al. [48] failed to find fungal correlates of obesity, with only *Debaryomyces* achieving a nominal *p* value < 0.05.

### 3.3. Secondary Outcomes

#### Microbial Taxonomic Signatures Associated with Obesity

Only two studies explicitly assessed taxonomic signatures associated with obesity [33,44]. In the Peters study [33], a microbiome-based machine learning model accurately classified obese persons and thus revealed an emerging taxonomic signature of obesity. Obesity was characterized by a higher abundance of class Bacilli and its families Streptococcaceae and Lactobacillaceae, and a lower abundance of several groups within the class Clostridia, including Christensenellaceae, Clostridiaceae, and Dehalobacteriaceae (q < 0.05). Their findings were consistent across two independent study populations. However, the Finucane study [44] concluded that there is no simple taxonomic signature of obesity in the microbiota of the human gut, since they found a large variability between studies (Human Microbiome Project (HMP) and MetaHIT) in the taxonomic composition of stool microbiomes that did not allow differences to be evident between lean and obese persons within studies and even across individuals. 

One study [56] classified obese and non-obese persons in two separate clusters, where 15/19 of the obese were classified in cluster 1 and all non-obese were fitted in cluster 2. The obese cluster showed reduced bacterial diversity compared to the non-obese cluster, a decreased B:F ratio, and an increased abundance of Proteobacteria. Moreover, in the obese microbiota cluster, *Clostridium* cluster *IV* and *XIVa* of the Firmicutes phylum were more abundantly present whereas Bacteroidetes phylum was less abundant.

Another study assessed enterotypes in obese persons and found that the Bact2 enterotype, which is characterized by a high proportion of *Bacteroides*, a low proportion of *Faecalibacterium* and low microbial cell densities, is more common in obese (17.7%) compared to non-obese persons (lean or overweight, 3.90%). However, obese persons who received statin treatment had a lower Bact2 prevalence (5.88%) [57].

### 3.4. Quality Assessment

A serious risk of bias was mainly found in the first two domains: confounding (*n* = 18), because studies did not adjust for potential confounders, and the selection of participants (*n* = 12), because nine studies recruited volunteers [17,19,35,37,45,46,47,52,58], two studies recruited participants through advertisement [53,56], and one study did not provide enough information on the recruitment process [54]. Of those studies of moderate risk for confounding, two studies did not adjust for variables but stratified results or excluded patients to minimize confounding [34,46], and 12 adjusted mainly for age and/or sex [20,32,33,37,43,45,48,49,51,53,55,60]. Other variables used for adjustment were dietary factors (e.g., intake of carbohydrates, vegetables excluding potatoes, intake of whole fruit, intake of whole grains) [33,48,53,55,60], batch effect [20], country [43], smoking status [49], BMI [48,49,55], study center [33,48], moderate-to-vigorous physical activity (MVPA) [48], diabetes [48], heath status [55], polyp status [33], visits returning to home country [48], education and income [48], and medications, including use of antibiotics and metformin [48]. Of the 32 studies, 11 were deemed as having an overall moderate risk of bias and 21 as having an overall serious risk of bias. In general, the meta-analyzed studies had a serious risk of bias (Table 1, Supplement Table S3).

## 4. Discussion

In the present systematic review, we found discrepant results on gut microbiome composition in obese versus non-obese persons across the studies. Statistically significant lower alpha diversity in obese versus non-obese persons was observed in a substantial proportion of studies, i.e., less than half for the Shannon index (nine out of 22 studies), half for the Simpson index (two out of four studies) and number of observed species/OTUs (four out of eight), and more than half for phylogenic distance (three out of four), and Chao1 (three out of five). No significant differences in obese versus non-obese persons were observed in meta-analysis of seven studies for the Shannon index. Microbial dissimilarities (beta diversity) were observed among BMI categories in the majority of the included studies. 

Observed differences in the gut microbiome composition at the phylum level in obese versus non-obese persons also showed discrepancies across studies. Firmicutes were found to be significantly higher in obese versus non-obese persons in six out of seventeen studies, whereas Bacteroidetes were found to be significantly lower in obese persons in four out of eighteen studies, but also significantly higher in four other studies. In the meta-analyses of six studies, Firmicutes were significantly higher and Bacteroidetes non-significantly lower in obese versus non-obese persons. The Firmicutes to Bacteroidetes ratio was found to be significantly higher in the obese in four out of ten studies, and the Bacteroidetes to Firmicutes ratio was found to be significantly lower in the obese in two out of three studies. The Bacteroidetes to Firmicutes ratio was statistically significantly lower in the obese versus non-obese in the meta-analysis of two studies, and a non-significantly higher Firmicutes to Bacteroidetes ratio was observed in the meta-analysis of two further studies. At the genus level, lower relative proportions of *Bifidobacterium* and *Eggerthella* were found in obese versus non-obese persons*,* whereas *Acidaminococcus*, *Anaerococcus*, *Catenibacterium*, *Dialister*, *Dorea*, *Escherichia-Shigella*, *Eubacterium*, *Fusobacterium*, *Megasphera*, *Prevotella*, *Roseburia*, and *Streptococcus*, and *Sutterella* were found to be higher in obese versus non-obese persons.

Our investigation shows no significant differences in the Shannon index between the obese and the non-obese in the meta-analysis, although in the qualitative synthesis a substantial proportion of studies showed statistically significantly lower alpha diversity in obese versus non-obese persons, and microbial dissimilarities (beta diversity) were observed among the BMI categories in the majority of the included studies, confirming that the microbial population abundances in the gut of obese and non-obese groups were distinct from each other. One previous meta-analysis [61] that included five studies using high-throughput technologies in the 16S rRNA gene and that were processed using a common computational pipeline, also showed a lack of a consistent alpha diversity trend (assessed by the Shannon index and number of observed species), likely due to the small effect size observed in studies investigating obesity compared to the larger effect size observed in studies on inflammatory bowel disease. Another previous meta-analysis [62] that re-analyzed the studies included in the Walters study [61] and added five more studies that included BMI and 16S rRNA gene sequence data found similar results to our observations. Only in two out of ten studies was there a significantly lower diversity in obese versus non-obese persons observed, whereas the rest showed similar trends—although these were non-significant. However, after applying a random-effects linear model to combine the studies, they found a statistically significantly lower richness, evenness, and diversity among obese persons (Shannon diversity index, observed richness, and Shannon evenness), despite the small effect size [62].

In the present systematic review, we found evidence that the composition of the gut microbiome of obese persons differs from the non-obese both at the phylum and genus level. In the qualitative synthesis at the phylum level, although the majority of the studies did not observe statistically significant differences in the relative abundance of the Gram-positive Firmicutes and Gram-negative Bacteroidetes between the obese and non-obese, a substantial proportion of studies observed statistically significantly higher Firmicutes (six out of seventeen) and lower Bacteroidetes (four out of eighteen) in obese versus non-obese persons. In the quantitative analysis, Firmicutes were significantly higher in the obese compared to the non-obese, whereas for Bacteroidetes non-significantly lower relative abundances were found. In both meta-analyses, significant heterogeneity was observed. 

In the qualitative synthesis at the phylum level, a statistically significantly higher F:B ratio and a lower B:F ratio was observed in obese persons compared to non-obese persons in a substantial number of studies, and a significantly lower B:F ratio was observed in the meta-analysis (two studies), whereas the F:B ratio was non-significantly higher (two further studies). Thus, no clear significant differences between the F:B or B:F ratio and obesity status was found. In animal studies, the F:B ratio is considered a hallmark of obesity and is therefore used as an outcome to assess the effects of many anti-obesity dietary supplements [63,64] based on their capacity to modulate the gut microbiome by reducing the F:B ratio in the obese. Two systematic reviews [24,25] that included studies measuring the ratio by high-throughput sequencing methods and other methods, such as culture, flow cytometry, and qPCR, among others, found more studies that showed no statistically significant differences in the F:B ratio between obese and non-obese persons than others that did, which is in agreement with our findings. 

Mechanistic explanations why Bacteroidetes and Firmicutes may be found at differential proportions in obese versus non-obese persons may be provided by studies investigating the host-gut microbiota relationship by relating the gut microbiome composition to circulating metabolites [50]. For example, short-chain fatty acids (SCFAs) are formed during the bacterial fermentation of carbohydrates in the colon, with acetate being the most common one found in the human colon, followed by butyrate and propionate [52,53]. A positive correlation between Firmicutes and fecal SCFA has been observed [53], which supports the hypothesis that obese persons have a distinct microbial profile that is more efficient in fermenting substrates and in producing higher fecal SCFA concentrations than that of their lean counterparts [52,53]. Whilst members of the Bacteroidetes phylum are acetate and propionate producers, bacteria belonging to the phylum of Firmicutes are mainly butyrate producers [65]. In animal studies acetate produced by the microbes is used as a substrate for the hepatic lipo- and gluconeogenesis, promoting obesity [28]. Diets rich in fats or carbohydrates favor the increase of Firmicutes and the decrease of Bacteroidetes both in animals and humans [28], which may also play a role in the differences in gut microbiome. However, dietary intake was considered only in a minority of the here included studies [33,48,53,55,60]. 

In the qualitative synthesis at the genus level, lower relative proportions of *Bifidobacterium* and *Eggerthella* (Actinobacteria phylum) were found in the obese group compared to the non-obese*,* whereas the genera of *Acidaminococcus*, *Anaerococcus*, *Catenibacterium*, *Dialister, Dorea, Eubacterium, Megasphera, Roseburia, Streptococcus* (Firmicutes phylum), *Fusobacterium* (Fusobacteria phylum)*, Prevotella* (Bacteroidetes phylum)*, Escherichia-Shigella*, and *Sutterella* (Proteobacteria phylum) were found to be significantly higher in the obese. The genera of *Sutterella* and *Catenibacterium* have been previously associated with obesity in children and adolescents [66] and pregnant women [67]. *Fusobacterium* and *Megasphera* produce butyrate from glutamate and lysine amino acids releasing harmful by-products like ammonia, which can have deleterious effects upon the host [65,68]. Discrepant results were found for the genera of *Akkermansia* (Verrucomicrobia phylum)*, Desulfovibrio* (Proteobacteria phylum)*, Alistipes, Parabacteroides, Bacteroides* (Bacteroidetes phylum)*, Anaerotruncus, Blautia, Clostridium, Coproccocus, Faecalibacterium, Oscillibacter, Oscillospira* and *Ruminoccocus* (Firmicutes phylum). These findings highlight the complexity of the microbial ecosystem of the gut and suggest that a greater relative abundance of the phylum Firmicutes and a lower relative abundance of Bacteroidetes in the obese do not necessarily translate into a common pattern of all genera belonging to these phyla, since several genera from the same phylum may be found in higher or in lower proportions in obese persons. For example, the Firmicutes genus *Oscillospira* was found to be significantly lower in obese compared to non-obese persons in four studies (and significantly higher in one study), which is in contrast to the hypothesis that obese gut microbiome is enriched in Firmicutes, that is also supported by our qualitative and quantitative analyses at the phylum level. One previous study meta-analyzing data from individuals with different health statuses found very few consistent genus-level associations between lean and obese persons [69]. For example, in agreement with our findings, they found that *Roseburia* was significantly enriched in obese persons [69]. Another previous meta-analysis found that, in agreement with our findings, *Bifidobacterium* was depleted in obese persons, whereas *Eubacterium rectale*, and *Roseburia intestinalis* were enriched in obese persons [61]. However, they found that *Bacteroides* were higher in obese whereas *Alistipes*, and *Oscillospira* were lower in obese compared to non-obese persons [61]. *Oscillospira* which has been found associated with leanness and health [70], has also been found to be enriched in metabolically healthy obese persons compared to metabolically unhealthy obese persons [6], likely due to their capability of producing high SCFA concentrations, like propionate and butyrate, which have beneficial effects on body weight control, inflammatory status, and insulin sensitivity [6,71]. We found no sufficient evidence of a specific taxonomic signature associated with obesity in the few studies addressing this question.

Overall, discrepancies found for each outcome when comparing obese with non-obese persons may be explained by factors such as sex and geographical differences, which may also reflect differences in their dietary habits. For example, Yasir et al. [35] compared the gut microbiome of obese and non-obese persons in France and Saudi Arabia with discrepant results, which may be likely due to, on the one hand, sex-differences, given that the French study population included both men and women whereas the Saudi population included only men, and, on the other hand, geographical differences in diet [35]. A large amount of study-specific variation can likely be attributed to differences in DNA isolation, sequencing and bioinformatic processing. Several studies have demonstrated that methodological differences can yield substantial variation in the results [72,73,74,75]. Our meta-analysis shows that technical variations may have masked potential biological differences when comparing across studies. The current method of analysis makes it very hard to make generic conclusions about the relation between microbiome and health and thereby cannot yet lead to medical grade interventions. Therefore, standardization and new analysis techniques are needed. An important factor that only recently has gained attention is the importance of including absolute counts, e.g., by applying spike-in standards [76] or flow cytometry based cell counting [77], to overcome the limitations of working with relative abundances. Additionally, some attempts have been made to unify several reference databases containing sequences of 16S rRNA genes to improve taxonomic classification [78]. Other factors that may contribute to discrepant findings are differences in the number and recruitment process of participants, which might preclude the observation of small differences between groups, as well as lack of adjustment for relevant lifestyle-associated factors that have an influence in the composition and diversity of the gut microbiome [27,61]. In order to increase the robustness of the available evidence, more individual-level meta-analyses contributing to the elucidation of the role of the gut microbiome in obesity are warranted, where the BMI comparison groups can be uniformly defined, and adjustment for confounders can be done in a comparable fashion covering critical factors influencing the gut microbiome composition, such as sex, age, diet, physical activity, and the microbiome data processed in a standardized manner [79].

### Strengths and Limitations

This is the first systematic review assessing the differences in the gut microbiome composition between obese and non-obese persons in a quantitative fashion through meta-analysis. Furthermore, for better comparability, we only included studies that analyzed the gut microbiome composition by means of high-throughput sequencing techniques. Furthermore, we were able to conduct meta-analyses for the two dominant phyla in the gut microbiome, Firmicutes and Bacteroidetes, although only a third of the included studies provided sufficient data. Nevertheless, it should be acknowledged that because many studies could not be included in the meta-analysis due to insufficient availability of data, our meta-analysis does not necessarily reflect the full available evidence. We conducted our searches in Pubmed and EMBASE, the two most commonly used databases for searching biomedical literature, although we cannot rule out that we may have missed some studies given that the search was restricted to the English language. In order to enable the inclusion of more published studies in meta-analysis, more standardized reporting is essential. In line with the limited data available for meta-analysis, we were unable to stratify analysis by country (as a proxy for diet) or sex, two known factors that can greatly influence the composition of the gut microbiome. Moreover, the definition of obese and non-obese groups was very heterogeneous, which may lead to an under- or over-estimation of the observed differences in our meta-analyses. The included studies were conducted in different countries with distinct eating habits that have an impact on the microbial ecology of the human gut microbiome, and were also of observational design, which means that confounding could have precluded small but significant differences in the composition of the gut microbiome between obese and non-obese persons. There are other factors, apart from the aforementioned sex [20,33,45], ethnicity or geographical location [20,34,35,43,48], and diet [9,41,45,47,53], that have the capacity to influence and therefore modify the composition of the gut microbiome, such as physical activity [45,58], smoking [47], and changes in the metabolites produced by gut bacteria [19,50,53,55], among other factors, e.g., fecal calprotectin, intestinal permeability [56], or cardiometabolic status [41,42]. These factors may explain the heterogeneity found among the meta-analyzed studies. However, we were not able to conduct a meta-regression analysis to examine whether these factors could explain the variability found in the meta-analyses. Moreover, the majority of the meta-analyzed studies were judged as having a serious risk of bias, downgrading the value of the level of evidence provided. Of the eleven meta-analyzed studies, six were not population-based (participants recruited through advertisements or as volunteers), and ten did not adjust for covariates. 

## 5. Conclusions

Overall, we observed discrepant findings in the gut microbiome composition of obese versus non-obese persons across studies both at the phylum and at the genus level. Lower alpha diversity in obese versus non-obese persons was observed in a substantial proportion of studies, but our meta-analysis on the Shannon Index yielded no significant differences. The B:F ratio might be considered as a marker of dysbiosis for obesity, although more research with standardized technologies is needed to consider it a hallmark of obesity or to replace it with a more fine-grained measure than comparing communities merely on the level of phyla. However, caution is required to infer these findings to any population given that few studies could be meta-analyzed and substantial heterogeneity was found. For the sake of data accuracy, reproducibility, and comparability of the results, standardization and harmonization of DNA extraction, amplification methods, high-throughput sequencing technologies and reference databases for taxonomic classification are needed to allow for better interoperability between studies, given that extensive omics data require more computational expertise and resources to manage and interpret results. More individual-level meta-analyses—preferably with standardized bioinformatics pipelines—allowing for more flexible and standardized approaches than study-level meta-analysis may help to clarify the here observed discrepant findings. Together with new studies using standardized and advanced methodologies this would allow for the design of disease prevention strategies, as well as personalized microbiome-modulating treatment strategies against obesity. 

## Figures and Tables

**Table 1:** Characteristics of the included studies.

Study ID	Country	Study Design Study Population (Recruitment Process and Ethnicity)	Sample Size (Age, Sex) ^1,2^	Obesity and Comparators (Definition, BMI Mean ± SD [Range]) ^1,2^	Stool Sample Collection Method and Storage	DNA Extraction Method (Region Amplified) Database Used	Sequencing Platform	RoB ^3,4^
Andoh 2016 [17]	Japan	Cross-sectional Volunteers	20 10 O; 10 L Age (31–58 y); 10 ♂ and 10 ♀	O: 38.1 ± 3.5 kg/m^2^ [35.7–49.2] L: 16.6 ±1.0 kg/m^2^) [14.2–17.7]	NR	FastPrep FP100A Instrument Primers used: 341 F and 806 R 16S (V3–V4) Techno-Suruga lab microbial identification Database DB-BA 9.0	Illumina MiSeq	S
Beaumont 2016 [37]	UK	Cross-sectional Healthy volunteers predominantly female twins from the TUK-D ^5^	1313: 496 MZ, 594 DZ and 223 unrelated individuals; average age 63 y (range 32–87); 96.4% ♀.	BMI; abdominal adiposity; VFM ^21^, (SFM ^22^, % pTF ^23^, AGR ^24^ and WHR ^25^). High and low groups: >1.5SD from the mean of the phenotype	Samples refrigerated or kept on ice for 1–2 days prior to arriving at the laboratory and stored max 8 weeks at −80 °C	DNA extraction method not reported Primers used: 515F and 806R 16S (V4). Greengenes May 2013 database	Illumina MiSeq	S
Borges 2018 [38]	Brazil	Cross-sectional Selected from a clinic nutrition service at the UFJF ^6^ teaching hospital and from the community (city of Juiz de Fora, MG, Brazil)	72 average age 39.61 y (range 18–60 y)	Eutrophic, OW, or O in accordance with their BMI	Collected in sterile vials given to the participants and sent immediately to the research laboratory for analysis	Fast DNA Spin Kit (MP Biomedicals, Illkirch, France). 18S rDNA using the universal primers (FungiQuant-F and FungiQuant-R) NCBI ^35^ nucleotide database	ABI Prism 3730 DNA sequencer (Applied Biosystems, San Francisco, CA, USA)	S
Borgo 2018 [18]	Italy	Cross-sectional Participants that underwent a screening colonoscopy for preventive purpose between January 2015 and January 2016 at the Department of the ASST Santi Paolo e Carlo of Milan	40 20 NW (♂: 48.7 y ± 10.2; ♀: 51:7 y ± 8.3); 20 O (♂: 53.8 y ± 7.7; ♀: 51.3 y ± 6.7)	O: BMI > 30 (35.8 ± 8.3) L: BMI < 25 (22.8 ± 1.8)	Collected 3 weeks after the colonoscopy and stored at −80 °C	QIAamp DNA Microbiome Kit (QIAGEN, Hilden, Germany). 16S (region NR). Greengenes bacterial database	Illumina MiSeq	S
Chavez-Carbajal 2019 [19]	Mexico	Cross-sectional Volunteer women from among people attending the Nutrition Clinic at the Universidad Iberoamericana in Mexico City	67 ♀ (25 control (mean age 23.3 y, SD 3.1), 17 obese (mean age 38.8, SD 8.4), 25 obese plus MetS (mean age 40.5 y, SD 10.3))	O class I: 30–34.99 kg/m^2^; O class II 35–39.99 kg/m^2^, O class III ≥ 40 kg/m^2^; NW: 18.5–24.99 kg/m^2^	Collected in a sterile stool container, aliquoted, and stored at −78 °C	ZR Fecal DNA MiniPrep™ (Zymo Research, Irvine, CA, USA) 16S (V3) Greengenes database (v 13.8)	316 Chip Kit v2 and the Ion Torrent PGM^TM^ System	S
Chen 2016 [20]	USA	Cross-sectional Mayo Clinic Biobank (patient medical records and patient-provided risk factor data) representative of the Midwestern US based on age, sex, race, BMI, smoking status and alcohol	118 ♂/♀ 58/60; Age ≥ 50 y 60/118	O: BMI ≥ 30 kg/m^2^ NO: BMI < 30 kg/m^2^	Collected by the subjects and returned to Mayo Clinic Rochester within 24 h, and stored at −80 °C	PowerSoil kit (MoBio, Carlsbad, CA, USA) according to the manufacturer’s instructions. 16S (V3–V5) RDP ^36^	Illumina MiSeq	M
Davis 2016 [40]	USA	Cross-sectional Randomly selected within the state of Alabama	81 Age (33 ± 13.3 y), ♀ (45/81, 56%)	BMI cut-offs not reported, but O, OW and N-UW mentioned in results; overall mean BMI in kg/m^2^ (28.3 ± 7.01)	Collected using the Fisher Scientific Commode Specimen Collection System and placed into a Fisher Scientific C & S ParaPak	Zymo ZR Fecal DNA MiniPrep™ Isolation Kit. 16S (V4) Database NR	Illumina MiSeq	S
Davis 2020 [39]	Australia	Cross-sectional (15th follow-up) Ongoing prospective cohort GOS ^7^ study. A random sample of adults reflecting the various cultural and socio-economic backgrounds in the region of South East Australia	158 Ages ranged from 34.2 to 92.2 y, with a mean of 65.9 y.	High FMI ^26^: FMI ^26^ ≥ 5.9 kg/m^2^ low FMI ^26^: FMI ^26^ < 5.9 kg/m^2^	Omnigene^®^ gut stool home collection. Samples were well mixed with the proprietary nucleic acid protective solution, aliquoted and frozen at −80 °C	QIAamp DNA mini kit (QIAGEN, Manchester, UK). Universal primers: 341-Forward and 806-Reverse 16S (V3–V4) Greengenes database	Illumina MiSeq	S
De la Cuesta-Zuluaga 2018 [41,42] (ref. [41] as the main study)	Colombia	Cross-sectional Adults enrolled in July–November 2014, with BMI ≥ 18.5 kg m^−2^, living in five Colombian cities. Participants were enrolled in similar proportions by BMI, city, sex and age	441 (NW: 138; OW 171; OB: 132) sex and age range (18–40 and 41–62 y). N (%), age (mean ± SD): NW healthy 91 (66%), 36.8 ± 10.6 y, 45.1%♂; NW abnormal 47 (34%), 43.3 ± 11.8 y, 46.8%♂: OW healthy 60 (35%), 38.4 ± 10.8 y, 43.3%♂: OW abnormal 111 (65%), 41.5 ± 10.9 y, 56.8%♂: O healthy 21 (16%), 43.1 ± 8.8 y, 28.6%♂: O abnormal 111 (84%), 42.7 ± 11.1 y, 48.6%♂	O: BMI ≥ 30.0 kg/m^2^ OW: 25.0 ≤ BMI < 30.0 kg/m^2^ NW: 18.5 ≤ BMI < 25.0 kg/m^2^	Sample in a hermetically sealed sterile receptacle, immediately refrigerated in household freezers and brought to a facility within 12 h and stored in dry ice and sent to a central laboratory	DNA was extracted using the QIAamp DNA Stool Mini Kit. Primers: F515 and R806 16 S (V4) Greengenes database (v 13.8)	Illumina MiSeq	S
Fei 2019 [43]	USA and African countries (Ghana, Jamaica and South Africa)	Cross-sectional African-origin adults (25–45 yrs) enrolled in METS ^8^ between 01/2010 and 12/2011 and followed yearly. Men and women from Ghana (*n* = 196), South Africa (*n* = 176), Jamaica (*n* = 92) and the US (*n* = 191) were collected in 2014	655 (L = 277; OW = 149; O = 229) Approx. 60% ♀. Aged 34.9 ± 6.4 y. Participants from South Africa and Jamaica were significantly younger than US participants (*p* < 0.001 and *p* = 0.016, respectively)	O: BMI ≥ 30 kg/m^2^ OW: 25.0 ≤ BMI < 30.0 kg/m^2^ L: BMI < 25 kg/m^2^ Results stratified by high and low WC ^27^: High WC ^27^ >102 cm in ♂ and >88 cm in ♀	Standard collection kit, brought to the site clinics and stored at −80 °C	DNeasy PowerSoil DNA Isolation Kit (Qiagen). 16 S (V4). Greengenes database (v 13.8)	Illumina MiSeq	M
Finucane 2014 [44]	USA (HMP study); Denmark and Spain (MetaHIT)	Cross-sectional HMP ^9^: Obese and non-obese adults living in Houston and Saint Louis; MetaHIT ^10^: A large survey of healthy obese and non-obese adults	HMP: (212 MetaHIT: 70)	HMP ^9^: O: BMI ≥ 30 L: BMI ≤ 25 MetaHIT ^10^: NR, just mentioned healthy O and NO adults	NR	DNA extraction method not reported. HMP ^9^: 16S (V1–V3 & V3–V5) MetaHIT ^10^: 16S GreenGenes database	HMP ^9^: Illumina GAIIx; MetaHIT ^10^: Illumina GA	S
Gallè 2020 [45]	Italy	Cross-sectional Students attending the University of Naples “Parthenope” and University of Rome “La Sapienza”	140 (UW: 7; NW: 106; OW: 24; O: 3) (48.6% ♂, mean age 22.5 ± 2.9 y)	BMI categories as defined by the WHO standards	Fecal swabs. Samples stored at 4–8 °C in a refrigerated container and were taken within 24 h	NR Shotgun metagenomics Greengenes database	Libraries for NGS ^39^ following the 16S Metagenomic Sequencing Library Preparation Guide (Illumina, San Diego, CA, USA)	S
Gao 2018 [46]	China	Cross-sectional Volunteers (information on recruitment NR)	551	WHO Asian BMI cut points O: ≥27.5 kg/m^2^; OW: 23–27.5 kg/m^2^; NW: 18.5–23 kg/m^2^; UW: <18.5 kg/m^2^	Self-collected by the volunteers using a 1.5 mL vial containing 1.0 mL inhibit EX Buffer. Samples shipped within 72 h of collection	QIAamp Fast DNA Stool Mini Kit (Qiagen, Stockach, Germany), following recommendations of the IHMS ^34^ guidelines. Universal primer set 341F/806R 16S (V3–V4). Greengenes database (v 13.8)	Illumina MiSeq	S
Harakeh 2020 [47]	Saudi Arabia	Cross-sectional Conducted between January 2015 and December 2015 on healthy adults of both genders, aged 18–55 years on students (including family members and friends) from King Abdulaziz University Medical campus	104 volunteers: UW = 21; NW = 31; OW = 28; O = 24 48% ♂ with median age ± IR was 24 ± 7.7 y	WHO criteria, BMI categories: O > 30 kg/m^2^, OW 25–30 kg/m^2^, NW 20–25 kg/m^2^, UW 18–20 kg/m^2^	Collected in aseptic conditions and immediately stored at −20 °C	NucleoSpin1 Tissue Mini Kit (Macherey Nagel, Hoerdt, France). 16S (V3–V4). SILVA123 SSU database	Illumina MiSeq	S
Kaplan 2019 [48]	USA	Cross-sectional HCHS/SOL a prospective, population-based cohort study of 16,415 Hispanic/Latino adults (aged 18–74 years, recruited in 2008–2011) selected using a two-stage probability sampling design from randomly sampled census block areas within four US communities (Chicago, IL; Miami, FL; Bronx, NY; San Diego, CA)	1674	O class I: 30–35 kg/m^2^ O class II: 35–40 kg/m^2^, O class III: >40 kg/m^2^; OW: 25–30 kg/m^2^, NW: 18.5 to 25 kg/m^2^	Plastic applicator into a supplied container with a stabilizer and 0.5-mm-diameter glass beads to mix stool and preservative. Aliquots frozen at −80 °C	Qiagen MagAttract PowerSoil DNA kit. 16S (V4) & ITS1 (Fungi). Greengenes 13_8 UNITE reference Database (Fungi)	Illumina MiSeq	M
Kasai 2015 [21]	Japan	Cross-sectional Subjects aged <65 years who had undergone colonoscopy at Mie Prefectural General Medical Center, Yokkaichi, Japan, between 2012 and 2013	56 (23 non-obese and 33 obese adults). Sex, male 30/56 (54%). Age mean ± SD (NO: 45.6 ± 9.6 y; O: 54.4 ± 8.2 y (*p* < 0.001))	O: ≥25 kg/m^2^, (*n* = 33) L: <20 kg/m^2^, (*n* = 23)	Collected prior to bowel preparation for colonoscopy. Stored at 4 °C after collection	MagDEA DNA 200 (GC) (Precision System Science). 16S (V3–V4). Apollon DB-BA database, ver 9.0 (TechnoSuruga Laboratory)	Illumina MiSeq	S
Loftfield 2020 [49]	Finland	Birth Cohort The NFBC 1966 included 12,055 expectant mothers within two Finnish provinces, Oulu and Lapland, with expected delivery dates during 1966. They were followed up to age 46 years	563; (Group 1: *n* = 167; Group 2: *n* = 167; Group 3: *n* = 163; Group 4: *n* = 66) 217 (38.5% ♂)	BMI at age 46 y: O: BMI ≥ 30 kg/m^2^ OW: 25.0 ≤ BMI < 30.0 kg/m^2^ NW: 18.5 ≤ BMI < 25.0 kg/m^2^	Collected at home, immediately frozen at −20 °C, brought to the study laboratory, and frozen without preservative in −70 °C	MO-BIO PowerSoil DNA isolation kit. barcoded 515F/806R primers 16S (V4) Greengenes database (v 13.8)	Illumina MiSeq	M
Oduaran 2020 [34]	South Africa	Cross-sectional Nested in the AWI-Gen project (part of the H3Africa ^11^). Recruited at two sites—the Bushbuckridge area within the Agincourt HDSS, Mpumalanga (rural) and Soweto, Johannesburg, Gauteng (urban)	170 HIV-negative women (51 at Soweto; 119 at Bushbuckridge). Age range of 43–72 y	O: BMI ≥ 30 kg/m^2^ OW: 25 ≤ BMI < 30 kg/m^2^ L: BMI < 25 kg/m^2^	DNA Genotek^®^’s OMNIgene microbial collection and stabilization kit and sent to the laboratory, aliquoted and frozen at −80 °C	DNA was extracted using Qiagen^®^’s QIAmp. 16S (V3–V4). RDP ^36^	Illumina MiSeq	M
Org 2017 [50]	Finland	Cross-sectional Ongoing population-based METSIM ^12^ study, a randomly selected cohort of unrelated men from the population register of Kuopio in Eastern Finland (pop. 95,000)	531 (aged 45–70 y)	O: BMI > 30 NO: BMI < 25	Collected during evaluation at the hospital and immediately stored at −80 °C	PowerSoil DNA Isolation Kit (MO BIO Laboratories, Carlsbad, CA, USA). 16S (V4) Greengenes database (v 13.8)	Illumina MiSeq	S
Osborne 2020 [51]	Bangladesh	Cross-sectional Ongoing, prospective HEALS ^13^ (11,746 married adults, 18–75 years old), recruited from October 2000 to May 2002. For the present study, randomly selected 400 HEALS ^13^ participants residing in 6 villages aged 25–50 y free from any major illness	250 mean age (mean ± SD): 48.6 ± 7.9 y 41% ♂	O: BMI ≥ 30 kg/m^2^ OW: 25.0 ≤ BMI < 30.0 kg/m^2^ NW: 18.5 ≤ BMI < 25.0 kg/m^2^ UW: BMI < 18.5 kg/m^2^	Collected in ThermoFisher Scientific vial, stored in a −20 °C freezer	MOBIO PowerSoil DNA Isolation Kit (MO BIO Laboratories, Carlsbad, CA, USA) 16S (V3–V4). Greengenes database (v 13.8)	Illumina MiSeq	M
Ozato 2019 [32]	Japan	Cross-sectional Iwaki Health Promotion Project launched in 2005 an annual health check-up for local residents living in the Iwaki region of Hirosaki City, Aomori Prefecture. Data obtained from the 2015 health checkup and a confirmation group from the 2016 health check (not in the 2015 one)	1001 (391 ♂, 610 ♀; mean age ± SD: 51.2 ± 14.1 y ♂, 54.2 ± 13.7 y ♀). Confirmation group: 326 (62% ♀, mean age 50.7 ± 17.5 y)	O: BMI ≥ 30 kg/m^2^ OW: 25 ≤ BMI < 30 kg/m^2^ NW: 20 ≤ BMI < 25 kg/m^2^ UW: BMI < 20 kg/m^2^ 4 groups: VFA ^28^ < 50, 50 ≤ VFA < 100, 100 ≤ VFA ^28^ < 150, VFA ^28^ ≥ 150	Collected using a commercial tube kit and cotton swabs within 3 days prior to the study, and stored at 4 °C	Bead-treated suspension using an automatic nucleic acid extractor (Precision System Science, Chiba, Japan). MagDEA DNA 200 (GC) reagent kit (Precision System Science) used for automatic nucleic acid. 16S (V3–V4). Database NR	Illumina MiSeq	M
Patil 2012 [52]	India	Cross-sectional Healthy individuals of Indian origin (21–62 years old) irrespective of gender	20 (5 individuals/group) Median age in years: L (age: 23 y), NW (age: 44 y), O (age: 45 y), O (treated) (age: 50 y)	O: 25–53 kg/m^2^, *n* = 5, O (treated): 25–36 kg/m^2^, regressing to normal BMI after SG ^29^ and AGB ^30^ surgeries. NW: 18–24 kg/m^2^, *n* = 5, L: BMI < 19 kg/m^2^, *n* = 5	Collected from unrelated healthy individuals. Stored at 4 °C and transported to laboratory on ice, and processed immediately or stored at −80 °C	QIAamp DNA Stool Mini Kit (Qiagen) with an additional step of bead beating using a mix of silica beads. 16S (V1–V4) RDP-II ^36^ database	ABI 3730 (Sanger sequencing)	S
Peters 2018 [33]	USA	Cross-sectional Two independent study populations based at colonoscopy clinics: the CDC ^14^ study, and the NYU ^15^ study. Predominantly white (94%)	599 (423 from CDU and 176 from NY study) Aged (62 ± 7 y)	O: BMI ≥ 30 kg/m^2^ OW: 25.0 ≤ BMI < 30.0 kg/m^2^ NW: 18.5 ≤ BMI < 25.0 kg/m^2^	Beckman Coulter Hemoccult II SENSA^®^ cards at home. Mailed to a laboratory for fecal occult blood testing. Samples refrigerated at 4 °C, and stored at −80 °C	PowerLyzer PowerSoil Kit (Mo Bio Laboratory Inc., CA) following manufacturer’s protocol. 16S (V4) Broad Institute Microbiome Utilities microbiomeutil-r20110519 database	Illumina MiSeq	M
Rahat-Rozenbloom 2014 [53]	Canada	Cross-sectional ♂ or non-pregnant, non-lactating ♀ aged > 17 years recruited via advertisements (University of Toronto campus) and from a pool of subjects previously involved in studies by their group	22 L (35.8± 4.2 y); OW (42.5 ± 3.9 y)	OW: BMI > 25 L: BMI ≤ 25	Plastic bag using the Fisher brand commode specimen collection system. Styrofoam box full of dry ice kept at −20 °C	DNA extraction method (Petrof EO et al., Microbiome 2013; 1:3) 16S (V6). Database NR	Ion Torrent sequencing	S
Salah 2019 [54]	Egypt	Cross-sectional Adult patients with obesity and diabetes in a population sample from El-Sharkia governate in North East Egypt	60; age (43.95 ± 13.35 y), gender (31 ♂, 29 ♀); 5 (C), 25 (O), 5 (D), and 25 (OD)	O: BMI 31 to 49 kg/m^2^ NW: 19–25 kg/m^2^	NR	QIAamp PowerFecal DNA Kit. 16S (V3–V4). SILVA SSU Ref NR dataset v.132 (OTUs), Greengenes (v 13.8), KEGG ^37^, KO ^38^ Database at level 2 and level 3	Illumina MiSeq	S
Thingholm 2019 [55]	Germany	Cross-sectional Individuals from the northern German cohorts PopGen ^16^ (*n* = 436) and FoCus ^17^ (*n* = 844)	1280 (L = 633; O = 494; OT2D = 153)	O: BMI >30 no T2D ^31^ OT2D: BMI >30 with T2D ^31^ L: BMI < 25 no T2D ^31^	NR	QIAamp DNA Stool Mini Kit from QIAGEN. 16S (V1–V2). RDP ^36^ database (16S) HUMAnN2 v0.11.1 (Shotgun)	Illumina MiSeq (16S); Illumina HiSeq (shotgun)	M
Verdam 2013 [56]	The Netherlands	Cross-sectional From May to September 2010, adults recruited through advertising at the Atrium Medical Center Parkstad in Heerlen, The Netherlands	28 Non-obese (*n* = 13): 9 lean and 4 were OW. Obese (*n* = 15): 9 were morbid obese. Aged 19–54 years. Sex F:M = 20:8	O: BMI range 30.5–60.3 kg/m^2^; morbid O: BMI > 40 kg/m^2^ (range 40.4–60.3 kg/m^2^); OW: BMI range 25.2–29.6 kg/m^2^; L: BMI range 18.6–24.6 kg/m^2^	Collected feces 24 h prior to the intestinal permeability test, kept refrigerated until test, and stored in aliquots at −20 °C	QIAamp Stool Kit by Qiagen 16S (region NR). HITChip specific database	HITChip, a phylogenetic profiling DNA microarray. Data extracted using the Agilent Feature Extraction	S
Vieira-Silva 2020 [57]	France Germany, Denmark	Cross-sectional BMIS ^18^ cohort was part of the overall MetaCardis recruitment (2013–2015) in several clinical departments ^19^	888 (NO (*n* = 414) versus O (*n* = 474)). Median age 54 [18–76} y; 574 ♀ and 314 ♂	O: BMI ≥ 30 NO: BMI < 30	Collected according to the IHMS ^34^ guidelines (modified SOP 04 V1 (collection without anaerobic bag)). Stored (less than 48 h) at −20 °C	DNA extracted following the IHMS guidelines (SOP 07 V2 H) Shotgun metagenomics NCBI ^35^ database (November 2016 version)	Ion proton system	S
Whisner 2018 [58]	USA	Cross-sectional Students from a larger study in two residence halls at Arizona State University in Tempe, Arizona (Fall 2014 and Spring 2015 semesters). 31.7% Hispanic; 39.0% White; 29.3% Other	82 (UW (5); NW (47); OW (18); O (12)). 57.3% ♀; age mean ± SD; 18.4 ± 0.6 y	O: BMI ≥ 30.0 kg/m^2^ OW: 25.0 ≤ BMI < 30.0 kg/m^2^ NW: 18.5 ≤ BMI < 25.0 kg/m^2^ UW: BMI < 18.5 kg/m^2^	Collection kit in small insulated cooler bags containing ice packs to be frozen immediately for 36–48 h in an insulated container. Samples delivered to the facility within 24 h of collection, and stored at −80 °C	PowerSoil DNA isolation kit as described by the manufacturer (MoBio Laboratories Ltd., Carlsbad, CA, USA) using a beadbeater (BioSpec, Bartlesville, OK, USA). 16S (V4). Greengenes database	Illumina MiSeq	S
Wilkins 2019 [59]	USA	Cross-sectional (retrospective) American Gut Project data (2012–2017)	600 (300 H; 300 O)	Chronic disease state: if “diagnosed” for CVD ^32^, diabetes, or KD ^33^, or with “obese” for BMI. Health status based on self-reported medical diagnoses. Healthy: “I do not have this condition” entry for diabetes, CVD ^32^, and KD ^33^, as well as “normal” for BMI	Samples collected (December 2012 and April 2017) from individuals from a global population	Method of DNA extraction NR 16S (region NR) Greengenes database (v 13.8)	NR	S
Yasir 2015 [35]	France and Saudi Arabia	Cross-sectional Volunteers from France and SA ^20^ living in urban areas	France: 28 (O (12) 58%♂; NW (16) 44%♂) SA ^20^: 18 (O (9); NW (9)). All ♂ Age mean ± SD: NW (France): 34 ± 5 y NW (SA ^20^): 28 ± 4 y O (France): 39 ± 13 y O (SA ^20^): 26 ± 3 y	O: BMI > 30 kg/m^2^ NW: BMI 20–25 kg/m^2^	Stool samples collected under aseptic conditions with clean, dry screw-top containers immediately stored at −20 °C	NucleoSpin Tissue Mini Kit (Macherey Nagel, Hoerdt, France). Primers: FwOvAd_341F and ReOvAd_785R 16S (V3–V4). SILVA SSU database	Illumina MiSeq	S
Yun 2017 [60]	Korea	Cross-sectional Kangbuk Samsung Health cohort study: men and women who underwent an annual or biennial examination at Kangbuk Samsung Hospital (June–September 2014)	1274 (NW (529); OW (326); O (419)); age 45.7 (9.0) y; sex, ♂: 63.7%	Revised Asia-Pacific BMI criteria by the WHO Western Pacific Region: O: BMI ≥ 25 OW: 23 ≤ BMI < 25 NW: 18.5 ≤ BMI < 23	NR	MO-BIO PowerSoil DNA Isolation Kit according to the manufacturer’s instructions. 16S (V3–V4). Greengenes database (v 13.8)	Illumina MiSeq	M

^1^ H: Healthy, L: Lean, NO: non-obese, NW: Normal weight, OW: Overweight, O: Obese, UW: underweight, WHO: world Health Organization; ^2^ ♂: males, ♀: females, MZ: monozygotic, DZ: dizygotic, SD: standard deviation; ^3^ RoB: Risk of Bias; ^4^ M: moderate risk of bias, S: serious risk of bias; ^5^ TUK-D: TwinsUK Adult Twin Registry; ^6^ UHFJF: University Hospital–Federal University of Juiz de Fora; ^7^ GOS: Geelong Osteoporosis Study; ^8^ METS: Modeling the Epidemiologic Transition Study; ^9^ HMP: Human Microbiome Project; ^10^ MetaHIT: METAgenomics of the Human Intestinal Tract; ^11^ H3Africa: Human, Heredity and Health in Africa consortium; ^12^ METSIM: METabolic Syndrome In Men; ^13^ HEALS: Health Effects of Arsenic Longitudinal Study; ^14^ CDC: the Centers for Disease Control and Prevention Study of In-home Tests for Colorectal Cancer; ^15^ NYU: New York University Human Microbiome and Colorectal Tumor study; ^16^ PopGEN: Population Genomic Diversity of Germany; ^17^ FoCus: Food Chain Plus; ^18^ BMIS: transnational Body Mass Index spectrum cohort; ^19^ departments of the Pitie-Salpetriere Hospital (Paris, France), the Integrated Research and Treatment Center for Adiposity Diseases (Leipzig, Germany), and the Novo Nordisk Foundation Center for Basic Metabolic Research (Copenhagen, Denmark); ^20^ SA: Saudi Arabia; ^21^ VFM: visceral fat mass; ^22^ SFM: subcutaneous fat mass, ^23^ pFT: % trunk fat, ^24^ AGR: android/gynoid ratio; ^25^ WHR: waist/hip ratio; ^26^ FMI: fat mass index; ^27^ WC: waist circumference; ^28^ VFA: visceral fat area where VFA ≥ 100 cm^2^ (obesity); ^29^ SG; ^30^ AGB:; ^31^ T2D: type 2 Diabetes; ^32^ CVD: cardiovascular diseases; ^33^ KD: kidney diseases; ^34^ IHMS: International Human Microbiome Standards guidelines; ^35^ NCBI: National Center for Biotechnology Information; ^36^ RDP: Ribosomal Database Project; ^37^ KEGGS: Kyoto Encyclopedia of Genes and Genomes; ^38^ KO: Kyoto Orthology Database; ^39^ NGS: next-generation sequencing. NR: not reported.

**Table 2:** Description of methods and results of microbial diversity and richness assessment.

Study ID (Author, Year)	Comparison Groups ^1^	Alpha Diversity Method ^2^	Alpha Diversity and Richness ^1,2^	Beta Diversity Method	Beta Diversity
Andoh 2016 [17]	O vs. L	Shannon index	Significantly lower in O (2.40 ± 0.17) vs. L (2.80 ± 0.14) *p* < 0.01	PCA ^3^	PCA ^3^ at phylum level showed different distribution of O and L peoples
Beaumont 2016 [37]	High vs. Low BMI	Shannon index	Significantly lower in high vs. low BMI (*p* = 0.0001)	—	—
Borges 2018 [38]	—	—	—	—	—
Borgo 2018 [18]	O vs. NW	Shannon index, observed species and Faith’s PD	Significantly lower α-diversity (PD) in O vs. *n* (*p* < 0.01). Shannon index and observed species are not reported by BMI group	Weighted and unweighted UniFrac ^4^ metrics and PCoA ^5^ Bray–Curtis distances	No separation was obtained between O and NW subjects (*p* > 0.05). Significant separation in LAM ^7^ samples between NW and O was observed
Chavez-Carbajal 2019 [19]	O vs. OMS vs. NW	Shannon index, Simpson index, Chao1, observed species.	Shannon index: no significant difference between groups O: 6.61 ± 0.36 OMS: 6.56 ± 0.38 NW: 6.32 ± 0.46 O vs. NW (*p* = 0.17) OMS vs. NW (*p* = 0.09) Simpson index: no significant difference between groups O: 0.97 ± 0.01 OMS: 0.97 ± 0.01 NW: 0.97 ± 0.02 O vs. NW (*p* = 0.28) OMS vs. NW (*p* = 0.52) Chao1 index: significantly higher in O vs. NW O: 787.1 ± 137.8 OMS: 769.4 ± 101.7 NW: 583.5 ± 87.8 OMS vs. NW (*p* = 0.003) O vs. NW (*p* = 0.002)	Unweighted UniFrac ^4^ analysis, PCoA ^5^	For the unweighted, PCoA ^4^ analysis clearly grouped the O and OMS separating them from the NW (ANOSIM ^8^; *p* = 0.01). Weighted analysis showed a similar result (ANOSIM ^8^, *p* = 0.01)
Chen 2016 [20]	—	Shannon index	NR by BMI groups	unweighted and weighted UniFrac ^4^ distances	NR by BMI groups
Davis 2016 [40]	O vs. OW vs. NW	Simpson’s Index of Diversity, Chao1, Shannon index Effective Number of Species	Shannon index Effective Number of Species: No significant differences reported O: 228.2 ± 134.1 OW: 218.1 ± 134.2 NW: 179.9 ± 103.1 *p* = not reported Chao1 and Simpson’s Index NR	—	—
Davis 2020 [39]	High FMI vs. Low FMI	Shannon index, Fishers index, Chao 1, Observed species	The alpha diversity and richness indices were lower in the high versus low FMI ^1^ group: Shannon index: no significant differences (data not shown) Fisher index: no significant differences (MD −6.2, 95%CI −12.7, 0.4; *p* = 0.065). Chao1 index: significantly lower in high vs. low FMI (MD −46.1, 95%CI −90.2, −2.0; *p* = 0.040); Observed species: significantly lower in high vs. low FMI (MD −46.1, 95%CI −86.5, −5.7; *p* = 0.026)	—	—
De la Cuesta-Zuluaga 2018 [41,42]	O vs. OW vs. L	Shannon index and number of observed OTUs	Shannon index: significant differences (lower in O/OW vs. L) O: 3.1 ± 0.7 OW: 3.0 ± 0.7 L: 3.2 ± 0.7 *p* = 0.04 # observed OTUs: significant differences (lower in O/OW vs. L) O: 142.5 ± 36.4 OW: 138.6 ± 35.6 L: 153.5 ±38.8 *p* = 0.002	Weighted and unweighted UniFrac ^4^ matrices (PERMANOVA ^6^)	NR by BMI groups
Fei 2019 [43]	O vs. OW vs. L	Shannon index, Chao1 diversity, observed OTUs	NR by BMI groups	Beta diversity (PERMANOVA ^6^)	NR by BMI groups
Finucane 2014 [44]	O vs. L	Shannon index, observed OTUs	Shannon index: No differences in O vs. L Richness (total number of OTUs): No difference in O vs. L	—	—
Gallè 2020 [45]	O/OW vs. NW/UW	Shannon index	No significant differences in O/OW (2.5 ± 0.2) vs. NW/UW (2.5 ± 0.2) *p* = 0.77	PCoA ^5^ using the METAGEN assist platform	ANOSIM ^8^ test yielded no significant dissimilarity for the BMI groups (R = −0.011, *p* = 0.5)
Gao 2018 [46]	O vs. NW	Shannon, Simpson, Number of observed OTUs	Shannon index: Significantly lower in O vs. NW (*p* < 0.01) Simpson index: Significantly lower in O vs. NW (*p* < 0.001) # observed OTUs: No significant difference in O vs. NW	PCoA ^5^ of samples by weighted and unweighted UniFrac ^4^ distance	Fecal microbial communities of the four BMI groups were not distinct from each other, indicating low among-group dissimilarities
Harakeh 2020 [47]	O vs. OW vs. NW vs. UW	Shannon index	No difference between UW, NW, OW and O individuals	—	—
Kaplan 2019 [48]	NW vs. OW vs. O (class I, II, III)	Shannon index	Significantly lower in O Class III vs. NW: Beta (95% CI) NW Ref. OW −0.01 (−0.08, 0.10) O Class I −0.08 (−0.18, 0.01) O Class II −0.09 (−0.21, 0.03) O Class III −0.19 (−0.35, −0.03)	Bray-Curtis distances	NR by BMI groups
Kasai 2015 [21]	O vs. L	Shannon index	Significantly higher in O vs. L *p* < 0.05	PCA ^3^	L subjects formed a cluster distinct from O subjects
Loftfield 2020 [49]	O vs. OW vs. NW	Shannon index, Faith phylogenetic diversity index (PD), and number of observed sequence variants	Shannon index, PD and number of observed sequence variants: No significant differences across groups.	Bray–Curtis and unweighted UniFrac ^4^; PCoA ^5^	Being O compared with normal BMI at age 46 was statistically significantly associated with Bray–Curtis, unweighted Uni-Frac, and weighted UniFrac distances (all *p* values ≤ 0.001); whereas OW BMI and BMI history were not statistically significantly associated with the beta diversity matrices
Oduaran 2020 [34]	O vs. L	Shannon index, Chao1	Shannon diversity in Bushbuckridge: No significant differences in O (4.56 ± 0.39) vs. L (4.49 ± 0.53 (4.56 ± 0.41 after exclusion of an outlier)) *p* = 0.85 Chao 1: Significantly higher in O vs. L (*p* = 0.001) Shannon diversity in Soweto: No significant differences in O (4.30 ± 0.56) vs. L (4.49 ± 0.34) *p* = 0.45 Chao 1: No significant differences in O vs. L (*p* = 0.33)	Bray-Curtis distances, PcoA ^4,5^	Beta diversity measurements showed statistically significant differences between the lean and obese groups in Bushbuckridge with calculated Bray-Curtis distances using the permutational analysis of variance (PERMANOVA ^6^) test (*p* = 0.02 for Bushbuckridge and *p* = 0.84 for Soweto)
Org 2017 [50]	—	Pielou’s index (evenness), and Fisher’s alpha (diversity)	NR by BMI groups	Bray–Curtis distance	NR by BMI groups
Osborne 2020 [51]	Tertile 1: 12.9–19.1 kg/m^2^; Tertile 2: 19.1–23.4 kg/m^2^; Tertile 3: 23.4–38.9 kg/m^2^	Shannon index, Chao1, number of observed OTUs	Shannon index: Significant decrease across BMI tertiles tertile 1: 4.6 ± 0.5 tertile 2: 4.5 ± 0.5 tertile 3: 4.4 ± 0.5 *p* < 0.01 Chao1: Significant decrease across BMI tertiles tertile 1: 10,848 ± 3916 tertile 2: 9761 ± 3006 tertile 3: 9162 ± 3590 *p* = 0.02 # observed OTUs: Non-significant decrease across BMI tertiles tertile 1: 3613 ± 1462 tertile 2: 3160 ± 999 tertile 3: 3093 ± 1317 *p* = 0.07	Unweighted UniFrac ^4^, weighted UniFrac ^4^, and Bray-Curtis distances	NR by BMI groups
Ozato 2019 [32]	High VFA vs. Low VFA	Shannon index	Significantly lower in High vs. Low VFA in men (*p* = 0.053) Non-significantly higher in High vs. Low VFA in women (*p* >0.05)	—	—
Patil 2012 [52]	O vs. NW	Shannon index and Simpson index	Shannon index: No significant differences reported O: 2.89 ± 0.56 NW: 2.84 ± 0.75 *p* = not reported Simpson index: No significant differences reported O: 0.11 ± 0.08 NW: 0.14 ± 0.18 *p* = not reported	UniFrac ^4^ analysis	Library cluster analysis clearly demonstrates clustering of lean and normal libraries except L3 (which has an unusually high Bacteroides genus counts). Interestingly, libraries O1 and O2 cluster in the normal/lean clade
Peters 2018 [33]	O vs. NW	Shannon index, Richness, and Evenness	Shannon index: Non-significantly lower in O vs. NW (beta = −0.11, *p* = 0.11, pHolm = 0.22) Evenness: Non-significantly lower in O vs. NW (beta = −0.01, *p* = 0.22, pHolm = 0.44). Richness (i.e., number of OTUs): Significantly lower in O vs. NW (beta = −9.87, *p* = 0.04, pHolm = 0.08); Significantly lower richness in O vs. NW in women (*p* = 0.03), but not in men (*p* = 0.47)	Weighted UniFrac^4^ distance, PCoA ^5^	Partial constrained analysis of PCoA ^4^ of the weighted UniFrac distance revealed separation of obese from both healthy-weight and OW participants on the main axis, with OW separated from healthy-weight participants on the secondary axis, although PCoA ^4^ did not reveal clustering by BMI category. In PERMANOVA ^6^ analysis of the weighted UniFrac distance, BMI category was not associated globally with overall microbiome composition (*p* = 0.14). In pairwise comparisons, overall microbiome composition differed between O and HW participants (*p* = 0.04, pHolm = 0.07), while OW and HW participants did not differ significantly (*p* = 0.64, pHolm = 0.64)
Rahat-Rozenbloom 2014 [53]	OW vs. L	Shannon index	No significant difference in OW (4.66) vs. L (4.92) *p* = 0.18	Weighted UniFrac ^4^ distances	PCoA ^5^ plots failed to reveal any difference in between the L and OW groups (data not shown)
Salah 2019 [54]	O vs. OD vs. NW	Shannon index, Number of OTUs	Shannon index: Significantly lower in O vs. NW (*p* < 0.01) Significantly higher in OD vs. NW (*p* < 0.05)- Number of OTUs: NR	PCoA ^5^ unweighted and weighted UniFrac ^4^ distance matrix	PCoA ^5^ plot based on unweighted Uni-Frac was built and showed significant BMI and diabetes-dependent clustering of samples (PERMANOVA ^6^; *p* = 0.001)
Thingholm 2019 [55]	O vs. L	PD calculated using the phylogenetic tree built on the aligned OTU sequences	PD significantly lower in O vs. L (*p* = 3.20310^−11^ by robust regression).	Function betadisper from the R package vegan with default settings to evaluate dispersion between groups	Composition (beta-diversity) of taxonomic and functional profiles (adonis q < 0.1), and taxonomic evaluation of dispersion (genera, betadisper q < 0.1) significantly lower in O vs. L, although not for functional features (betadispersion q > 0.1)
Verdam 2013 [56]	O vs. NO	Simpson’s reciprocal index of diversity (1/D)	Significantly lower in O (128.7 ± 33.2) vs. NO (174.6 ± 37.3) *p* = 0.002	—	—
Vieira-Silva 2020 [57]	—	Observed richness was calculated using phyloseq	NR by BMI groups	PCoA ^5^ using Bray–Curtis dissimilarity with Hellinger transformation	NR by BMI groups
Whisner 2018 [58]	—	PD metrics calculated by QIIME via Faith’s PD	NR by BMI groups	PCoA ^5^ using weighted and unweighted UniFrac ^4^ distances	NR by BMI groups
Wilkins 2019 [59]	—	—	—	Weighted UniFrac ^4^ beta-diversity	NR by BMI groups
Yasir 2015 [35]	O vs. NW	Shannon Index Chao Index Number of OTUs	Shannon Index, Chao Index, and Number of OTUs reported at OTU cutoffs of 3, 6 and 9 distance units France O significantly lower diversity and richness than NW at all the OTU cutoffs (*p* < 0.05). Saudi Arabia No significant difference in diversity and richness between O and NW at all the OTU cutoffs	PCoA ^5^ calculated in QIIME by choosing Bray–Curtis distance methods at the genus level	PCoA ^5^ showed that O and NW individuals clustered independently. NW individuals from France and Saudi Arabia clustered together, but O Saudis clustered independently from obese French
Yun 2017 [60]	O vs. OW vs. NW	PD metrics calculated by QIIME ^9^	Significantly lower diversity (PD) in O vs. NW (*p* < 0.01) and OW vs. NW (*p* < 0.01)	PCoA^5^ of weighted UniFrac ^4^	Weighted UniFrac ^4^ PCoA ^5^ identified significant differences between groups (ANOSIM ^8^; R = 0.020, *p* = 0.001)

^1^ L: Lean; NO: non-obese; NW: Normal weight; OW: Overweight; O: Obese; OD: obese diabetic; OMS: Obese and metabolic syndrome; UW: underweight; VFA: visceral fat area; FMI: fat mass index; ^2^ PD: phylogenetic distance; OTUs: operational taxonomic units; ^3^ PCA: Principal component analysis; ^4^ UniFrac: unique fraction metric; ^5^ PCoA: Principal Coordinates analysis; ^6^ PERMANOVA: permutational multivariate analysis of variance ^7^ LAM: lumen-associated microbiota; ^8^ ANOSIM: Analysis of Similarities; ^9^ QIIME: Quantitative Insights Into Microbial Ecology. NR: not reported.

**Table 3:** Differences in the relative abundance of bacteria at phylum level.

Study ID (Author, Year)	Comparison Groups ^1^	Actinobacteria ^1^	Bacteroidetes ^1^	Firmicutes ^1^	Fusobacterium ^1^	Proteobacteria ^1^	Verrucomicrobia ^1^	Other	B/F ^2^ or F/B ^3^ Ratio ^1^
Andoh 2016 [17]	O vs. L	No significant differences in O vs. L	No significant differences in O vs. L O: 31.2 ± 14.1% L: 32.9 ± 6.4% *p* = 0.38	Significantly higher in O vs. L O: 42.6 ± 8.5% L: 35.1 ± 5.2% *p* = 0.018	Significantly higher in O vs. L O: 1.86 ± 4.20% L: 0.00 ± 0.00% *p* = 0.002	No significant differences in O vs. L	—	Unclassified (*p* > 0.05)	B/F ^1^ ratio O: 0.86 ± 0.63 L: 0.96 ± 0.27 Not significant
Beaumont 2016 [37]	—	—	—	—	—	—	—	—	—
Borges 2018 [38]	—	—	—	—	—	—	—	—	—
Borgo 2018 [18]	O vs. NW	No significant differences in O vs. NW O: 1.5 ± 1.2 NW: 1.4 ± 1.9	No significant differences in O vs. NW O: 38.5 ± 12.7NW: 41.7 ± 7.9	No significant differences in O vs. NW O: 53.8 ± 11.3 NW: 51.2 ± 8.1	—	No significant differences in O vs. NW O: 3.5 ± 2.7 NW: 4.5 ± 5.7	No significant differences in O vs. NW O: 2.4 ± 5.3 NW: 0.9 ± 1.7	—	—
Chavez-Carbajal 2019 [19]	O + MetS vs. O vs. NW	No significant differences between groups O: 1.27% O + MetS: 1.29% NW: 2.32% *p* = 0.1667	No significant differences between groups O: 22.50% O + MetS: 23.43% NW: 36.20% *p* = 0.7125	Significantly higher in O vs. NW O: 72.97% O + MetS: 73.34% NW: 56.95% *p* = 0.0029	—	No significant differences between groups O: 2.80% O + MetS: 1.45% NW: 4.20% *p* = 0.1160	—	Includes Verrucomicrobia, Spirochaetes and Fusobacteria. O: 0.22% O + MetS: 0.37% NW: 0.14% *p* < 0.0001	F/B^2^ ratio O + MetS: 3.13 O: 3.24 C: 1.57 *p* = not reported (significance not reported)
Chen 2016 [20]	O vs. NW	—	No significant differences in O vs. NW O: 4.339 × 10^−1^ NW: 5.004 × 10^−1^ q = 0.080	No significant differences in O vs. NW O: 5.226 × 10^−1^ NW: 4.660 × 10^−1^ q = 0.080	No significant differences in O vs. NW O: 1.433 × 10^−2^ NW: 1.446 × 10^−3^ q = 0.080	—	—	Chrisiogenetes: O: 1.927 × 10^−5^ NW: 1.680 × 10^−4^ q = 0.080	—
Davis 2016 [40]	—	—	—	—	—	—	—	—	—
Davis 2020 [39]	—	—	—	—	—	—	—	—	—
De la Cuesta-Zuluaga 2018 [41,42]	O vs. OW vs. NW	—	Significantly lower in O vs. NW O: 8.2 ± 14.1 OW: 10.8 ± 17 NW:11.5 ± 19.8 *p* = 0.04	No significant differences between groups O: 53.9 ± 30.9 OW: 51.8 ± 29.5 NW: 48.5 ± 20.8 *p* = 0.62	—	—	—	—	Significantly lower in O vs. NW B/F ^1^ ratio O: 0.15 ± 0.28 OW: 0.22 ± 0.42 NW: 0.23 ± 0.40 *p* = 0.04
Fei 2019 [43]	—	—	—	—	—	—	—	—	—
Finucane 2014 [44]	O vs. L	No differences	No differences (*p* = 0.30)	No differences (*p* = 0.86)	No differences	No differences	—	No differences	—
Gallè 2020 [45]	O/OW vs. NW/UW	—	No significant differences in O/OW vs. NW/UW O/OW: 31.8 ± 8.9 NW/UW: 33.4 ± 10.4 *p* = 0.54	No significant differences in O/OW vs. NW/UW O/OW: 61.1 ± 8.7 NW/UW: 58.9 ± 13.1 *p* = 0.47	—	—	—	—	No significant differences in O/WO vs. NW/UW F/B ^2^ ratio O/OW: 2.2 ± 1.3 NW/UW: 2.1 ± 1.1 *p* = 0.56
Gao 2018 [46]	O vs. OW vs. NW vs. UW	No differences in O vs. UW	Significantly higher in O vs. UW (*p* < 0.05)	No differences in O vs. UW	Significantly higher in O vs. UW (*p* < 0.01)	Significantly higher in O vs. UW (*p* < 0.05)	—	—	—
Harakeh 2020 [47]	O vs. NW	—	—	—	Significantly lower in O vs. NW (*p* = 0.005, FDR = 0.014)	—	—	—	—
Kaplan 2019 [48]	—	—	—	—	—	—	—	—	—
Kasai 2015 [21]	O vs. NO	No differences between groups O: 8.0 ± 7.1% NO: 8.2 ± 6.7% *p* = 0.917	Significantly lower in O vs. NO O: 37.0 ± 14.0% NO: 44.0 ± 9.8% *p* = 0.033	No differences between groups O: 40.8 ± 15.0% NO: 37.0 ± 9.1% *p* = 0.241	O: 1.58% NO: 0.07% *p* > 0.05	O: 0.91% NO: 1.20% *p* > 0.05	—	Increase in the proportion of “unclassified” phyla (O 21.76% vs. NO 8.54%) were observed in the O group relative to the NO group Sinergistetes: O: 0.00% NO: 0.03%	Significantly higher in O vs. UW F/B ^2^ ratio O: 1.7 ± 1.7 NO: 0.9 ± 0.4 *p* = 0.045
Loftfield 2020 [49]	O vs. NW	—	Significantly higher in O vs. NW	—	—	—	—	—	No significant differences in O vs. NW F/B ^2^ ratio expressed as beta coefficients OW vs. NW: −29.7 (*p* = 0.26) O vs. NW: 4.66 (*p* = 0.88)
Oduaran 2020 [34]	O vs. L	No differences between groups (*p* > 0.05)	Significantly higher in O vs. L (*p* < 0.05)	No differences between groups (*p* > 0.05)	No differences between groups (*p* > 0.05)	No differences between groups (*p* > 0.05)	No differences between groups (*p* > 0.05)	No differences between groups (*p* > 0.05)	—
Org 2017 [50]	—	—	—	—	—	—	—	—	—
Osborne 2020 [51]	—	—	—	—	—	—	—	—	—
Ozato 2019 [32]	G1: BMI < 20 G2: 20 ≤ BMI < 25 G3: 25 ≤ BMI < 30 G4: BMI ≥ 30	No differences between groups	Significant decrease across BMI groups in women (*p* for trend: <0.001). No significant association across BMI groups in men	Significant increase across BMI groups in women (*p* for trend: 0.004). No significant association across BMI groups in men	—	No differences between groups	—	—	—
Patil 2012 [52]	O vs. L	No differences between groups	No differences between groups	No differences between groups	—	No differences between groups	—	Unclassified: No differences between groups	—
Peters 2018 [33]	O vs. OW vs. NW	—	Not associated with BMI category (*p* = 0.49)	Not associated with BMI category (*p* = 0.40)	—	—	—	—	F/B ^2^ ratio (Kruskal-Wallis test *p* = 0.94). No results per group provided
Rahat-Rozenbloom 2014 [53]	O/OW vs. L	—	No significant differences in O/OW (6.4 ± 4.3) vs. L (19.4 ± 6.1) *p* = 0.335	Significantly higher in O/OW (83.1 ± 4.1) vs. L (69.5 ± 5.8) *p* = 0.008	—	—	—	—	Significantly higher in O/OW vs. LF/B ^2^ ratio O/OW: 34.3 ± 1.6 L: 6.8 ± 1.0 (*p* = 0.023, or *p* = 0.0098 when adjusted for age). F/B ^2^ ratio expressed as a base 2 logarithm derived from the median center log-ratio-transformed values of each sample.
Salah 2019 [54]	O vs. OD vs. D vs. NW	Significant differences: NW: 0.54% O: 0.69% D: 0.77% OD: 1.52% *p* = 0.04	Non-significant differences: NW:34.25% O: 44.94% D: 38.4% OD: 37.16% *p* = 0.07	Non-significant differences: NW. 36.4% O: 48.72% D: 49.1% OD: 51.09% *p* = 0.31	Non-significant differences: NW: 0% O: 0% D: 0% O + D: 0.48 *p* = 0.20	Significant differences NW: 24.65% O: 5.61% D: 11.1% OD: 7.48% *p* = 0.02	Significant differences NW: 3.86% O: 0.006% D: 0.43% OD: 1.54% *p* < 0.001	Significant differences: Euryarchaeota (*p* < 0.001) Lentisphaerae (*p* = 0.01) Synergistetes (*p* < 0.001) Tenericutes (*p* = 0.01)	No significant differences reported: F/B ^2^ ratio NW = 1.06 O: 1.08 OD: 1.37 *p* = not reported
Thingholm 2019 [55]	—	—	—	—	—	—	—	—	—
Verdam 2013 [56]	O vs. NO	—	Significantly lower in O vs. NO O: 5.9% ± 5.8% NO: 19.2% ± 9.2%; *p* < 0.002	Significantly higher in O vs. NO O: 85.8% ± 8.5% NO: 74.6% ± 9.2%; q = 0.002	—	Several members of the Proteobacteria including those related to *E. aerogene*, *K. pneumoniea*, *Vibrio*, and *Yersina* spp. were positively associated with BMI and more abundantly present in obese	—	—	B/F ^1^ ratio strongly decreased in O (*p* = 0.0002).
Vieira-Silva 2020 [57]	—	—	—	—	—	—	—	—	—
Whisner 2018 [58]	BMI < 18.5 BMI 18.5–24.9 BMI 25.0–29.9 BMI ≥ 30.0	—	—	—	—	—	—	—	F/B ^2^ ratio did not differ by BMI *p* = 0.413 No results per group provided
Wilkins 2019 [59]	—	—	—	—	—	—	—	—	—
Yasir 2015 [35]	O vs. NW (France) O vs. NW (Saudi Arabia)	No significant differences (France and SA)	Significantly higher in O vs. NW (France) (*p* = 0.05) No significant differences (SA)	No significant differences (France) Significantly higher in O vs. NW (SA) (*p* = 0.001)	—	Significantly higher in O vs. NW (France) (*p* = 0.002) No significant differences (SA ^4^)	No significant differences (France and SA)	—	—
Yun 2017 [60]	O vs. OW vs. NW	—	—	—	—	—	—	—	No significant differences in F/B ^2^ ratio. No results per group provided

^1^ D: diabetes; G1–4; Groups 1–4; L: lean; NO: non-obese; NW: normal weight; O: obese; OD: obesity and diabetes; O+MetS: obesity and metabolic syndrome; OW: overweight; UW: underweight; ^2^ B/F ratio: Bacteroidetes to Firmicutes ratio; ^3^ F/B ratio: Firmicutes to Bacteroidetes ratio; ^4^ SA: Saudi Arabia. N.A.: not available.

**Table 4:** Significant differences in the relative abundance of bacteria at genus level between obese and non-obese persons.

Genus (#Studies)	Significantly Higher in Obese		Significantly Lower in Obese	
	*n*	[Citations]	*n*	[Citations]
Firmicutes				
*Acetanaerobacterium* ^1^	1	[34]	—	—
*Acidaminococcus*	3	[32,48,51]	—	—
*Anaerococcus*	2	[17,59]	—	—
*Anaerotruncus*	1	[45]	1	[48]
*Blautia*	4	[20,33,49,50]	1	[32]
*Butyrivibrio* ^1^	—		1	[54]
*Catenibacterium*	2	[34,48]	—	—
*Clostridium* ^2^	1	[54]	1	[35]
*Clostridium_IV* ^1^	1	[34]	—	—
*Clostridium_XIVa* ^3^	1	[34]	—	—
*Clostridium_XIVb* ^3^	1	[34]	—	—
*Coprobacillus*	—		1	[48]
*Coproccocus*	3	[17,19,59]	1	[33]
*Dehalobacterium*	—		1	[33]
*Dialister*	2	[18,19]	—	—
*Dorea* ^4^	5	[20,35,43,45,49]	—	—
*Eubacterium*	2	[20,45]	—	—
*Faecalibacterium* ^2^	2	[19,54]	2	[17,35]
*Finegoldia*	—	—	1	[17]
*Fusicatenibacter* ^3^	—	—	1	[34]
*Gemella*	1	[45]	—	—
*Intestinimonas*	1	[34]	—	—
*Lachnoanaerobaculum*	—	—	1	[17]
*Lachnobacterium*	1	[45]	—	—
*Lachnospira*	1	[19]	—	—
*Lactobacillus* ^2^	1	[35]	—	—
*Megasphera*	2	[43,48]	—	—
*Oscillibacter* ^3^	1	[34]	1	[55]
*Oscillospira*	1	[37]	4	[18,33,48,51]
*Parvimonas*	1	[17]	—	—
*Phascolarctobacterium* ^1^	1	[34]	—	—
*Roseburia*	4	[19,43,49,54]	—	—
*Ruminoccocus* ^3^	4	[34,37,41,49]	2	[48,54]
*Sporobacter* ^3^	1	[34]	—	—
*Staphylococcus*	1	[54]	—	—
*Streptococcus* ^3^	5	[33,34,43,45,48]	—	—
*Subdoligranulum*	—	—	1	[17]
Bacteroidetes				
*Alistipes* ^1^	2	[17,34]	1	[55]
*Bacteroides* ^2^	3	[35,52,59]	2	[17,19]
*Parabacteroides* ^1,3^	2	[34,43] ^1^	1	[34] ^3^
*Paraprevotella*	—	—	1	[45]
*Prevotella*	3	[34,48,54]	—	—
Actinobacteria				
*Bifidobacterium*	—	—	1	[32]
*Corynebacterium*	1	[59]	—	—
*Eggerthella* ^5^	—	—	2	[48,60]
*Olsenella*	—	—	1	[17]
*Rothia*	1	[59]	—	—
Fusobacteria				
*Fusobacterium*	2	[17,46]	—	—
*Sneathia*	1	[45]	—	—
Proteobacteria				
*Desulfovibrio*	1	[43]	1	[17]
*Escherichia-Shigella* ^2,3^	2	[34,35]	—	—
*Haemophilus* ^3^	1	[34]	—	—
*Jannaschia*	1	[45]	—	—
*Oxalobacter* ^3^	1	[34]	—	—
*Paucibacter*	1	[45]	—	—
*Succinivibrio*	1	[45]	—	—
*Sutterella* ^1^	2	[34,46]	—	—
Verrucomicrobia				
*Akkermansia* ^1^	1	[34]	2	[54,55]
Synergistetes				
*Cloacibacillus*	—	—	1	[48]
Euryarchaeota				
*Methanobrevibacter*	—	—	1	[50]
Lentisphaerae				
*Victivallis* ^3^	—	—	1	[34]

^1^ Ref. [43]: in Bushbuckridge; ^2^ Ref. [57]: in France; ^3^ Ref. [43]: in Soweto; ^4^ Ref. [57]: in Saudi Arabia; ^5^ Ref. [56]: no longer significant after adjustment for carbohydrate.

## Data Availability

Not applicable.

## Supplementary Materials

The following are available online at https://www.mdpi.com/article/10.3390/nu14010012/s1, Table S1: Search strategies, Table S2: Description and decision criteria for each domain in ROBINS-I, Table S3: Critical appraisal of the included studies using the ROBINS-I tool, Figure S1: Included studies that reported alpha diversity using Shannon index, amplified region V3–V4, and Greengenes database for taxonomic classification; Figure S2. Forest plot of the differences in alpha diversity between obese and non-obese stratified by Shannon and Simpson indices after sensitivity analysis.
