# Supplementary material for: Gut Microbiome Composition in Obese and Non-Obese Persons: A Systematic Review and Meta-Analysis
Source: Nutrients. 2021 Dec 21;14(1):12. doi: 10.3390/nu14010012 (PMC8746372; doi:10.3390/nu14010012)

Supplement Material

Persons: A Systematic Review and Meta-Analysis

Mariona Pinart [1], Andreas Dötsch [2], Kristina Schlicht [3], Matthias Laudes [3,4], Jildau Bouwman [5], Sofia K. Forslund [6,7,8,9,10,11], Tobias Pischon [1,10,11,12] and Katharina Nimptsch [1,*]

[1] Molecular Epidemiology Research Group, Max Delbrück Center for Molecular Medicine in the Helmholtz Association (MDC), 13125 Berlin, Germany; Mariona.PinartGilberga@mdc-berlin.de (M.P.); tobias.pischon@mdc-berlin.de (T.P.)
[2] Department of Physiology and Biochemistry of Nutrition, Max Rubner-Institut (MRI)—Federal Research Institute of Nutrition and Food, 76131 Karlsruhe, Germany; andreas.doetsch@mri.bund.de
[3] Institute of Diabetes and Clinical Metabolic Research, University of Kiel, 24105 Kiel, Germany; Kristina.Schlicht@uksh.de (K.S.); matthias.laudes@uksh.de (M.L.)
[4] Division of Endocrinology, Diabetes and Clinical Nutrition, Department of Internal Medicine 1, Kiel University, 24118 Kiel, Germany
[5] Microbiology and Systems Biology Group, Toegepast Natuurwetenschappelijk Onderzoek (TNO), Utrechtseweg 48, 3704 HE Zeist, The Netherlands; jildau.bouwman@tno.nl
[6] Experimental and Clinical Research Center, A Cooperation of Charité-Universitätsmedizin Berlin and Max Delbrück Center for Molecular Medicine, Lindenberger Weg 80, 13125 Berlin, Germany; Sofia.Forslund@mdc-berlin.de
[7] Charité-Universitätsmedizin Berlin, Corporate Member of Freie Universität Berlin, Humboldt-Universität zu Berlin, Berlin Institute of Health, 10117 Berlin, Germany
[8] Host-Microbiome Factors in Cardiovascular Disease Lab, Max Delbrück Center for Molecular Medicine in the Helmholtz Association (MDC), 13125 Berlin, Germany
[9] Structural and Computational Biology Unit, European Molecular Biology Laboratory, 69117 Heidelberg, Germany
[10] Biobank Core Facility, Berlin Institute of Health at Charité-Universitätsmedizin Berlin, 10178 Berlin, Germany
[11] German Centre for Cardiovascular Research (DZHK), Partner Site Berlin, 10785 Berlin, Germany
[12] Biobank Technology Platform, Max Delbrück Center for Molecular Medicine in the Helmholtz Association (MDC), 13125 Berlin, Germany
* Correspondence: Katharina.Nimptsch@mdc-berlin.de; Tel.: +49-30-9046-4573

Supplement Table S1. Search strategies.

Pubmed (from inception to 19 Nov 2020)

Search	Query	Results
**#4**	Search: ((anthropometry[Title/Abstract] OR "body weight"[Title/Abstract] OR obesity[Title/Abstract] OR obese[Title/Abstract] OR overweight[Title/Abstract] OR adiposity[Title/Abstract] OR "body mass"[Title/Abstract] OR BMI[Title/Abstract] OR "waist circumference"[Title/Abstract] OR "abdominal fat"[Title/Abstract] OR "body fatness"[Title/Abstract] OR"body size"[Title/Abstract] OR "body fat distribution"[Title/Abstract] OR "waist-to-hip ratio" [Title/Abstract] OR "waist hip ratio"[Title/Abstract] OR adiposity [Title/Abstract]) AND ("gastrointestinal microbiome"[MeSH Terms] OR microbiome)) AND ((observational study) OR (cohort) OR (cross-sectional) OR (case-control) OR (longitudinal) OR (follow/up) OR (prospective))	**947**
#3	Search: (observational study) OR (cohort) OR (cross-sectional) OR (case-control) OR (longitudinal) OR (follow/up) OR (prospective)	3919070
#2	Search: "gastrointestinal microbiome"[MeSH Terms] OR microbiome<br>"gastrointestinal microbiome"[MeSH Terms] OR "microbiome s"[All Fields] OR "microbiomic"[All Fields] OR "microbiomics"[All Fields] OR "microbiota"[MeSH Terms] OR "microbiota"[All Fields] OR "microbiome"[All Fields] OR "microbiomes"[All Fields]	82231
#1	Search: anthropometry[Title/Abstract] OR "body weight"[Title/Abstract] OR obesity[Title/Abstract] OR obese[Title/Abstract] OR overweight[Title/Abstract] OR adiposity[Title/Abstract] OR "body mass"[Title/Abstract] OR BMI[Title/Abstract] OR "waist circumference"[Title/Abstract] OR "abdominal fat"[Title/Abstract] OR "body fatness"[Title/Abstract] OR"body size"[Title/Abstract] OR "body fat distribution"[Title/Abstract] OR "waist-to-hip ratio" [Title/Abstract] OR "waist hip ratio"[Title/Abstract] OR adiposity [Title/Abstract]	716018

EMBASE via Ovid (from 1947 to 19 Nov 2020)

Search	Query	Results
#1	(anthropometry or "body weight" or obesity or obese or overweight or adiposity or "body mass"or BMI or "waist circumference" or "abdominal fat" or "body fatness" or "body size" or "body fatdistribution" or " waist-to-hip ratio "or" waist hip ratio "or adiposity) .mp.	1472509
#2	("gastrointestinal microbiome" or "microbiome gastrointestinal microbiome" or "microbiome s" or"microbiomic" or "microbiomics" or "microbiota" or "microbiota" or "microbiome" or"microbiome"). mp.	89322
#3	("observational study" or "cohort" or "cross-sectional" or "case-control" or "longitudinal" or"follow-up" or "follow up" or "follow / up" or "prospective"). mp.	4502098
**#4**	1 and 2 and 3	**1940**

mp = title, abstract, heading word, drug trade name, original title, device manufacturer, drug manufacturer, device trade name, keyword, floating subheading word, candidate term word

Supplement Table S2. Description and decision criteria for each domain in ROBINS-I.

Domain	Explanation	Judgements
**Bias due to confounding**	Is there potential for confounding of the effect of exposure in this study? Did the authors use an appropriate analysis method that controlled for all the important confounding domains (i.e. age, sex, race/ethnicity)?<br><br>Notes: Confounding is expected in all observational studies.	<u>Low risk of bias:</u> No bias expected due to confounding.<br><br><u>Moderate risk of bias:</u> Confounding is expected: age, sex, and race/ethnicity have been appropriately controlled for in a multivariable-adjusted analysis *or* the authors statistically investigated whether the confounding domains have an effect on the risk estimate and excluded the confounder from the multivariable model if there was no effect on the overall effect estimate.<br><br><u>Serious risk of bias:</u> At least one known important domain was not measured or appropriately controlled for.<br><br><u>No information:</u> No information on which confounder have been controlled for.
**Bias due to selection of participants**	Was selection of participants truly representative of the average in the target population (all subjects or random sampling)? Were adjustment techniques used that are likely to correct for the presence of selection biases?<br><br>Notes: In observational studies, it is unlikely that post-exposure variables influenced selection of participants into the study. Exclusion of participants may be mostly based on missing data, which will be considered in the domain referring to missing data (see below).	<u>Low risk of bias:</u> All participants were selected from the population using random sampling methods and the response rate is not lower than 80%.<br><br><u>Moderate risk of bias:</u> Selection into the study was somewhat representative of the average in the target population. * (nonrandom sampling).<br><br><u>Serious risk of bias:</u> Participants were selected by means of a convenient sample (no sampling strategy performed)<br><u>No information:</u> No information is reported about selection of participants into the study.
**Bias due to exposure assessment**	Were exposure groups clearly defined and adequately assessed? Was the information used to define the exposure groups based on reasonable a priori data?<br><br>Notes: Bias introduced by either differential or non-differential misclassification of exposure status.	<u>Low risk of bias:</u> Exposure status was well defined (comprehensibly derived categories); *and* no measurement error is expected in its assessment.<br><br><u>Moderate risk of bias:</u> Exposure status is well defined (comprehensibly derived categories); *and* exposure was measured

	Non-differential misclassification is unrelated to the outcome and will usually bias the estimated effect of exposure towards the null. Differential misclassification occurs when misclassification of exposure status is related to the outcome or the risk of the outcome, and is likely to lead to bias.	subjectively (i.e. self-reported height and weight).<br><br>Serious risk of bias: Exposure status is not well defined;<br><br>No information: No definition of exposure or no explanation of the source of information about exposure status is reported.
**Bias due to missing data**	Were there missing outcome data? Were participants excluded due to missing data on exposure status? Were participants excluded due to missing data on other variables needed for analysis?<br><br>Notes: Missing data on exposure variables and other variables are expected to be missing at random and not related to exposure or outcome.	Low risk of bias: Data on exposure and outcome variables were reasonably complete (<10% missing data) and was unlikely to introduce bias;<br><br>Moderate risk of bias: There is a proportion of missing outcome (microbiome) data (between 10% and 50%) in the original cohort independent of exposure (obesity);<br><br>Serious risk of bias: High proportions (>50%) of missing outcome (microbiome) data; or moderate proportions (between 10% and 50%) of missing outcome data dependent of exposure (obesity) or no information about whether missings in outcome data depend on exposure<br><br>No information: No information is reported about missing data or the potential for data to be missing.
**Bias due to measurement of the outcome**	Could the outcome measure have been influenced by knowledge of the exposure status? Were the methods of outcome assessment comparable across exposure groups? Was any systematic error in measurement of the outcome related to exposure status?<br><br>Notes: In observational studies, it is not expected that outcome assessors were aware of exposure status of the participants.	Low risk of bias: The methods of outcome assessment were comparable across exposure groups; *and* the outcome measure was unlikely to be influenced by knowledge of the exposure status of study participants; *and* any error in measuring the outcome is unrelated to exposure status (i.e. objective measures such as confirmed medical records, record linkage).<br><br>Moderate risk of bias: The methods of outcome assessment were comparable across exposure groups; *and* any error in measuring the outcome may be minimally related to exposure status.

		<u>Serious risk of bias:</u> The methods of outcome assessment were not comparable across exposure groups;<br>*or* an error in measuring the outcome was related to exposure status.<br><br><u>No information:</u> No information is reported about the methods of outcome assessment.
**Bias due to selective reporting of the results**	Is the reported effect estimate likely to be selected from multiple analyses of exposure-outcome relationship?<br>Is the reported effect estimate likely to be selected from different subgroups?<br><br>Notes: In observational studies, it is unusual to publish an a priori analysis plan or protocol.	<u>Low risk of bias:</u> There is a clear description of all analysis and the analyses are consistent and all reported results correspond to all intended outcomes, analyses and sub-cohorts.<br><br><u>Moderate risk of bias:</u> The analyses are clearly defined; *and* there is indication of selection of the reported analysis from among multiple analyses; *and* there is indication of selection of the cohort or subgroups for analysis and reporting on basis of the results (e.g. estimates not shown for all analyses).<br><br><u>Serious risk of bias:</u> There is a high risk of selective reporting from among multiple analyses; *or* the cohort or subgroup is selected from a larger study for analysis and appears to be reported based on the results.<br><br><u>No information:</u> There is too little information to make a judgement.
**Overall judgement**	Low risk of bias	The study is judged to be at low risk of bias for all domains.
	Moderate risk of bias	The study is judged to be at low or moderate risk of bias for all domains.
	Serious risk of bias	The study is judged to be at serious risk of bias in at least one domain, but not at critical risk in any domain.

Supplement Table S3. Critical appraisal of the included studies using the ROBINS-I tool.

Study ID	Confounding	Selection of participants	Exposure assessment	Missing data	Measurement of the outcome	Selective reporting of the results	Overall judgement
**Andoh 2016**	Serious	Serious	Serious	NI	Low	Low	Serious
**Beaumont 2016**	Moderate	Serious	Low	Moderate	Low	Low	Serious
**Borges 2018**	Serious	Moderate	Serious	NI	Low	Low	Serious
**Borgo 2018**	Serious	Moderate	Low	NI	Moderate	Low	Serious
**Chavez-Carvajal 2019**	Serious	Serious	Low	NI	Low	Low	Serious
**Chen 2016**	Moderate	Moderate	Low	NI	Low	Low	Moderate
**Davis 2016**	Serious	Low	Serious	NI	Low	Low	Serious
**Davis 2020**	Serious	Moderate	Low	NI	Low	Low	Serious
**De la Cuesta-Zuluaga 2018 a and b**	Serious	Moderate	Low	NI	Low	Low	Serious
**Fei 2019**	Moderate	Moderate	Low	NI	Low	Low	Moderate
**Finucane 2014**	Serious	Moderate	Low	NI	Low	Low	Serious
**Gallè 2020**	Moderate	Serious	Moderate	NI	Moderate	Low	Serious
**Gao 2018b**	Moderate	Serious	Moderate	NI	Low	Low	Serious
**Harakeh 2020**	Serious	Serious	Moderate	NI	Low	Moderate	Serious
**Kaplan 2019**	Moderate	Moderate	Moderate	Low	Low	Low	Moderate
**Kasai 2015**	Serious	Moderate	Moderate	NI	Low	Low	Serious
**Loftfield 2020**	Moderate	Moderate	Low	Moderate	Low	Low	Moderate
**Oduaran 2020**	Moderate	Moderate	Low	Moderate	Low	Low	Moderate
**Org 2017**	Serious	Moderate	Low	NI	Low	Low	Serious
**Osborne 2020**	Moderate	Low	Low	Low	Low	Low	Moderate
**Ozato 2019**	Moderate	Moderate	Low	Low	Low	Low	Moderate
**Patil 2012**	Serious	Serious	Low	NI	Moderate	Low	Serious
**Peters 2018**	Moderate	Moderate	Moderate	Low	Low	Low	Moderate
**Rahat-Rozenbloom 2014**	Moderate	Serious	Low	NI	Low	Low	Serious
**Salah 2019**	Serious	Serious	Moderate	NI	Low	Low	Serious
**Thingholm 2019**	Moderate	Moderate	Moderate	Moderate	Low	Low	Moderate
**Verdam 2018**	Serious	Serious	Moderate	NI	Moderate	Low	Serious
**Vieira-Silva 2020**	Serious	Moderate	Low	NI	Moderate	Low	Serious
**Whisner 2018**	Serious	Serious	Low	Moderate	Low	Low	Serious
**Wilkins 2019**	Serious	Moderate	Moderate	NI	Moderate	Low	Serious
**Yasir 2015**	Serious	Serious	Moderate	NI	Low	Low	Serious
**Yun 2017**	Moderate	Moderate	Low	NI	Low	Low	Moderate

NI: No information

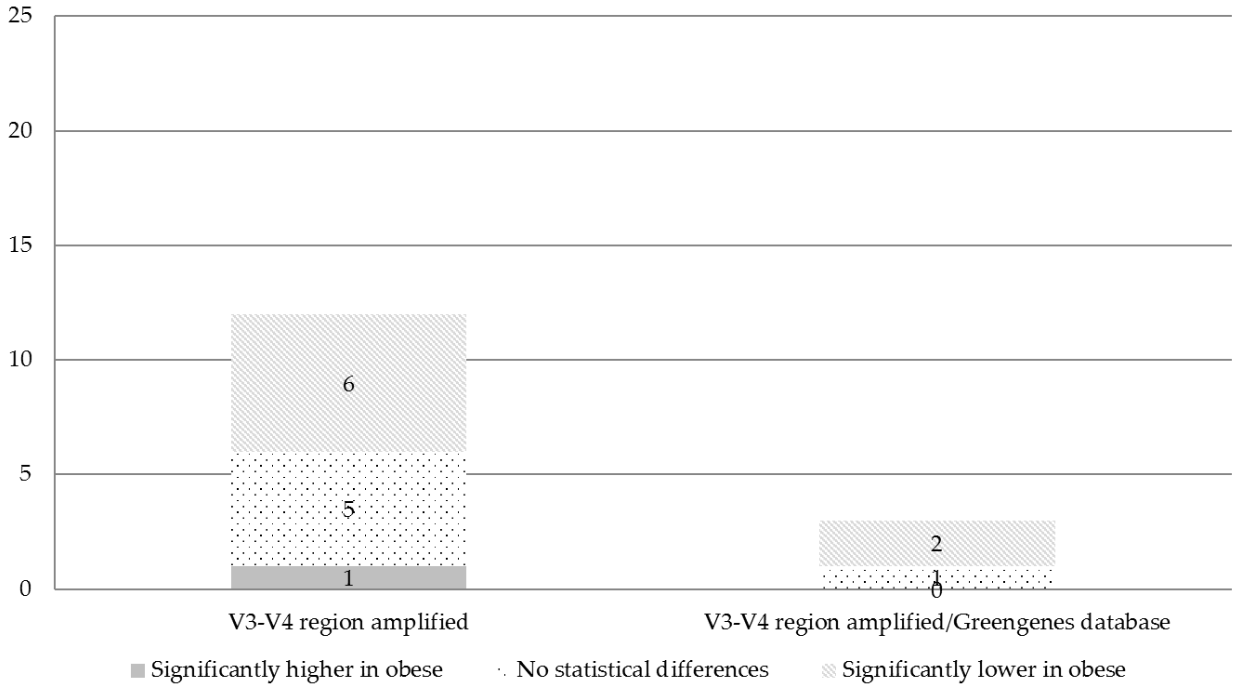

Supplementary Figure S1. Number of included studies that reported alpha diversity using Shannon index as significantly higher (grey), lower (diagonal stripes) or not different (dotted) when comparing obese to non-obese persons, amplified region V3-V4, and used Greengenes database for taxonomic classification.

Supplement Figure S2. Forest plot of the differences in alpha diversity between obese and non-obese stratified by Shannon and Simpson indices after sensitivity analysis.

Study	Experimental Total	Experimental Mean	Experimental SD	Control Total	Control Mean	Control SD	MD	95%-CI
Chavez-Carvajal 2019	17	6.61	0.36	25	6.32	0.46	0.29	[0.04; 0.54]
De la Cuesta-Zuluaga 2018	132	3.10	0.70	138	3.20	0.70	-0.10	[-0.27; 0.07]
Gallè 2020	27	2.50	0.20	113	2.50	0.20	0.00	[-0.08; 0.08]
Oduaran 2020_Bushbuckridge	66	4.56	0.41	21	4.49	0.53	0.07	[-0.18; 0.32]
Oduaran 2020_Soweto	40	4.30	0.56	9	4.49	0.34	-0.19	[-0.47; 0.09]
Patil 2012	5	2.89	0.56	10	2.84	0.75	0.04	[-0.63; 0.72]
**Random effects model**	**287**			**316**			**0.01**	**[-0.11; 0.13]**

Heterogeneity: $I^2 = 43\%$, $p = 0.12$

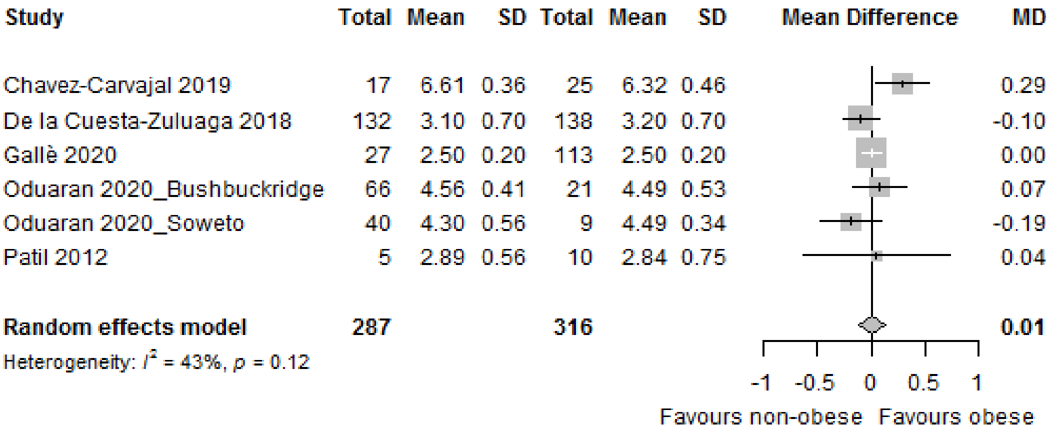

Supplement: Supplementary file 1 [file nutrients-14-00012-s001.zip › nutrients-1511510-supplementary.pdf]
